# Chemical Composition and Biological Activities of *Pelargonium* sp.: A Review with In Silico Insights into Potential Anti-Inflammatory Mechanism

**DOI:** 10.3390/molecules30153198

**Published:** 2025-07-30

**Authors:** Diana Celi, Karina Jimenes-Vargas, António Machado, José Miguel Álvarez-Suárez, Eduardo Tejera

**Affiliations:** 1 Facultad de Ingeniería y Ciencias Aplicadas, Universidad de Las Américas (UDLA), Quito 170504, Ecuador; 2 Bio-Cheminformatics Research Group, Universidad de Las Américas (UDLA), Quito 170504, Ecuador; 3 Departament of Computer Science and Information Technologies, Faculty of Computer Science, Universidade da Coruña, Campus Elviña s/n, 15071 A Coruña, Spain; 4 Centro de Biotecnologia dos Açores (CBA), Departamento de Biologia, Faculdade de Ciências e Tecnologia, Universidade dos Açores, 9500-321 Ponta Delgada, Portugal; 5 Laboratorio de Bacteriología, Instituto de Microbiología, Colegio de Ciencias Biológicas y Ambientales COCIBA, Universidad San Francisco de Quito (USFQ), Quito 170901, Ecuador; 6 Laboratorio de Investigación en Ingeniería en Alimentos (LabInAli), Departamento de Ingeniería en Alimentos, Colegio de Ciencias e Ingenierías, Universidad San Francisco de Quito (USFQ), Quito 170901, Ecuador; 7 Laboratorio de Bioexploración, Colegio de Ciencias Biológicas y Ambientales, Universidad San Francisco de Quito (USFQ), Quito 170901, Ecuador

**Keywords:** *Pelargonium* species, flavonoids, target-based predictive models, in silico analysis, anti-inflammatory response

## Abstract

The *Pelargonium* genus, encompassing over 280 species, remains markedly underexplored despite extensive traditional use for respiratory, gastrointestinal, and dermatological disorders. This review of aqueous, alcoholic, and hydroalcoholic extracts reveals critical research gaps: only 10 species have undergone chemical characterization, while 17 have been evaluated for biological activities. Phytochemical analysis identified 252 unique molecules across all studies, with flavonoids emerging as the predominant class (n = 108). Glycosylated derivatives demonstrated superior bioactivity profiles compared to non-glycosylated analogs. Phenolic acids (n = 43) and coumarins (n = 31) represented additional major classes. Experimental studies primarily documented antioxidant, antibacterial, and anti-inflammatory effects, with emerging evidence for antidiabetic, anticancer, and hepatoprotective activities. However, methodological heterogeneity across studies limits comparative analysis and comprehensive understanding. In silico target prediction analysis was performed on 197 high-confidence molecular structures. Glycosylated flavonols, anthocyanidins, flavones, and coumarins showed strong predicted interactions with key inflammatory targets (ALOX15, ALOX5, PTGER4, and NOS2) and metabolic regulators (GSK3A and PI4KB), providing mechanistic support for observed therapeutic effects and suggesting potential applications in chronic inflammatory and metabolic diseases. These findings underscore the substantial therapeutic potential of underexplored *Pelargonium* species and advocate for systematic research employing untargeted metabolomics, standardized bioassays, and compound-specific mechanistic validation to fully unlock the pharmacological potential of this diverse genus.

## 1. Introduction

The *Pelargonium* genus, the second largest in the Geraniaceae family, comprises approximately 280 species predominantly native to southern Africa. These species are renowned for their aromatic properties, ornamental value, and diverse medicinal applications, positioning them as valuable resources in the fragrance, cosmetics, and pharmaceutical industries. The ethnobotanical significance of *Pelargonium* is particularly pronounced in southern Africa, where various plant parts have been integrated into traditional healing practices, culinary traditions, and cultural ceremonies for centuries [1,2,3].

Among the most notable species, *P. sidoides* and *P. reniforme* have a long-standing history in southern African medicine, particularly in the treatment of respiratory conditions. Their root extracts, often prepared as infusions or decoctions, are used to manage symptoms of bronchitis, tuberculosis, asthma, and sinusitis. These remedies, recognized for their anti-inflammatory properties, have historically been employed to alleviate coughs, colds, and bronchitis, as well as to address tuberculosis [4,5,6].

While respiratory applications represent the most documented traditional uses, the therapeutic potential of *Pelargonium* extends considerably beyond these applications to encompass various gastrointestinal disorders. Several species, including *P. antidysentericum*, *P. cucullatum*, *P. triste*, *P. reniforme*, and *P. betulinum*, have been traditionally used to address a variety of ailments, such as colic, gastric disorders, diarrhea, dysentery, and nephritis. The roots and herbs of these plants are commonly prepared as decoctions or consumed directly to alleviate symptoms [7]. For example, in the Namaqualand region of southwestern Africa, *P. antidysentericum* is used to treat dysentery, with its tuber incorporated into milk decoctions [1], while *P. endlicherianum* has shown anthelmintic properties, with its roots typically prepared as decoctions to expel roundworms and fresh herbs consumed for effective treatment [8].

In addition to gastrointestinal applications, *Pelargonium* species demonstrate significant dermatological properties. *P. grossularioides* has been used topically to treat eczema, while *P. cucullatum* has been applied to open sores and wounds. Additionally, *P. triste* is employed in the treatment of hemorrhoids and sore throats. The leaves and roots of these plants are often prepared as poultices or applied directly to affected areas to reduce inflammation and promote healing [9].

Beyond their medicinal applications, *Pelargonium* species also have culinary significance. In certain cultural contexts, they are used as flavoring agents. For example, *P. tomentosum*, with peppermint-scented leaves, imparts a refreshing flavor to teas and desserts [10]. In the culinary traditions of the Western Balkan region, *P. graveolens* is used as a flavoring agent in various foods and beverages, including alcoholic drinks and pink-colored products [11].

Despite the extensive traditional use and growing commercial interest in *Pelargonium* species, comprehensive scientific documentation of their phytochemical diversity and biological activities remains limited. Most research has concentrated on a few well-known species, particularly *P. sidoides*, leaving the vast majority of the genus underexplored. This knowledge gap represents a significant opportunity for drug discovery and validates the need for systematic investigation. Therefore, this review aims to synthesize existing literature on the phytochemical composition and bioactive potential of *Pelargonium* species while employing computational approaches to predict molecular targets and elucidate potential mechanisms of action. By integrating traditional knowledge with modern analytical techniques and bioinformatics tools, this work seeks to provide a foundation for future research and highlight the untapped therapeutic potential within this diverse genus.

## 2. Results

### 2.1. Chemical Composition

Chemical characterization studies revealed significant underexploration of the *Pelargonium* genus despite its rich diversity. From 176 articles initially identified through systematic searching, only 18 studies met the stringent criteria for comprehensive chemical characterization using advanced analytical techniques (HPLC, MS, and NMR). The majority of excluded studies focused on clinical applications, extraction optimization, or general phytochemical screening without detailed molecular identification.

The chemical analysis was further constrained by the predominant focus on essential oil research within the genus. While essential oils have been extensively studied for their aromatic monoterpenes and sesquiterpenes (primarily citronellol, geraniol, and linalool) [12,13], the non-volatile chemical diversity present in aqueous, alcoholic, and hydroalcoholic extracts—which are most relevant to traditional medicinal uses—remains largely unexplored.

### 2.2. Distribution of Studies on Pelargonium Species, Countries, and Plant Parts

**Figure 1:**
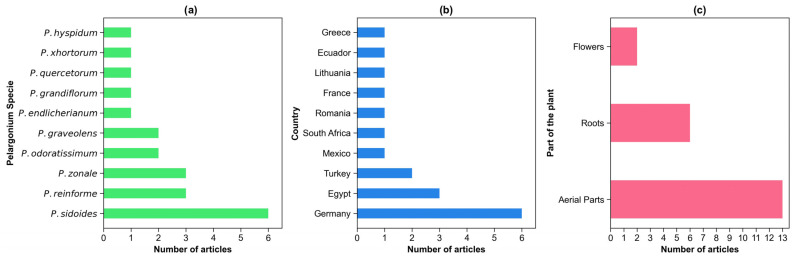
Summary of the distribution of the 18 reviewed studies on *Pelargonium* species. (**a**) Number of publications per species focused on chemical composition. (**b**) Number of studies per country. (**c**) Plant part analyzed, categorized into aerial parts (stems and leaves), roots, and flowers.

Research distribution across *Pelargonium* species revealed significant taxonomic bias within the available literature. Despite the genus encompassing over 280 species predominantly native to southwestern Africa, only 10 species have undergone comprehensive chemical characterization. *P. sidoides* emerged as the most extensively studied species with six publications, followed by *P. reniforme* and *P. zonale* with three studies each (Figure 1a). The remaining species were represented by only one or two studies, indicating substantial gaps in our understanding of the genus’s chemical diversity.

Germany emerged as the leading country in *Pelargonium* research, contributing six publications (Figure 1b). These studies, conducted at the Free University of Berlin, in collaboration with Dr. Willmar Schwabe GmbH & Co. KG (Kaslu, Germany), primarily focused on *P. sidoides* and *P. reniforme*. The research initially sought to identify the South African plant source used for treating respiratory diseases, originally attributed to *P. reniforme*. Through detailed chemical composition analyses and the identification of novel compounds, primarily using LC-MS-MS, these studies identified *P. sidoides* as the species possessing the desired medicinal properties [14].

Notably, despite the African origin and extensive traditional use of *Pelargonium* species, only Egypt and South Africa contributed to chemical characterization studies, representing a significant geographic research gap. This absence of African research institutions in phytochemical studies contrasts sharply with the continent’s rich ethnobotanical knowledge of the genus.

The aerial parts of *Pelargonium* species, including stems and leaves, have been the primary focus of chemical studies, with 13 articles dedicated to this topic (Figure 1c). This preference likely stems from the ease and sustainability of collecting aerial parts, as well as their prominence in traditional medicine [15,16,17]. Roots followed with six articles, while flowers were the least studied, appearing in only two publications.

Roots play a particularly significant role in the ethnobotanical use of *Pelargonium*. Despite their significant role in traditional medicine, including uses of *P. cucullatum* for respiratory ailments, *P. luridum* for gastrointestinal disorders, and *P. botulinum* for wound healing, roots have been comparatively under-investigated in chemical studies [5]. These findings highlight the medicinal value of tuberous roots, which are often consumed raw, boiled, or roasted. Despite being the second-most studied plant part, roots have received comparatively less attention than aerial parts, potentially overlooking important bioactive compounds.

### 2.3. Analytical Techniques and Chemical Diversity

Three analytical approaches of increasing sophistication were employed across the reviewed studies. Basic chromatographic methods (HPLC-UV and HPLC-DAD) were used for four species (*P. zonale*, *P. odoratissimum*, *P. quercetorum*, and *P. hispidum*), relying on retention time and UV spectral comparison against known standards. While reliable for well-characterized compounds, this approach was the sole method used for *P. odoratissimum* and *P. hispidum*, highlighting the need for more comprehensive analytical strategies.

Advanced mass spectrometric techniques (HPLC-MS, UPLC-MS, and LC-MS) were employed in nine studies, particularly for *P. sidoides* and *P. reniforme*, enabling detection of structurally complex metabolites, including highly oxidized coumarins, O-galloyl-C-glycosyl flavones, pelargoniins, and ellagitannins. The most comprehensive studies combined HPLC-MS with nuclear magnetic resonance (NMR) spectroscopy, providing both sensitive detection and definitive structural elucidation. This integrated approach proved essential for accurate metabolite profiling and reliable interspecies comparisons.

A total of 252 unique molecules were identified and standardized using SMILES notation. Database curation classified compounds based on identification confidence: 219 molecules received high-confidence designation (“0”) through direct database matching (PubChem and J-GLOBAL) or explicit structural description; 21 molecules were classified as ambiguous (“1”) due to non-standard nomenclature requiring interpretation; 12 molecules were assigned uncertain status (“2”) with manual structural approximations to preserve chemical space diversity.

**Figure 2:**
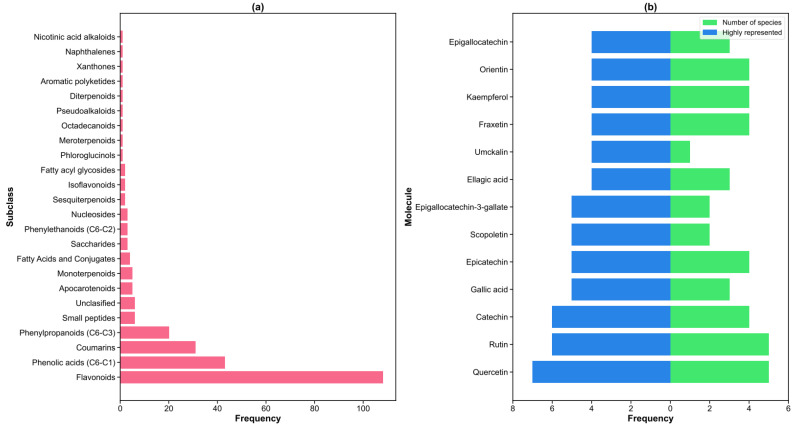
Results of chemical composition. (**a**) Distribution of molecular subclasses in all *Pelargonium* species according to NPClassifier. (**b**) Highly represented molecules across different publications (blue bars) and number of species in which the molecule was reported (green bars).

NPClassifier analysis revealed flavonoids as the dominant chemical class (n = 108), followed by phenolic acids (n = 43), coumarins (n = 31), and phenylpropanoids (n = 20) (Figure 2a). Classes with fewer than six molecules were grouped as rare subclasses, indicating the concentrated nature of *Pelargonium*’s chemical diversity within specific biosynthetic pathways.

Figure 2a shows the subclass distribution of 252 molecules, according to NPClassifier. Flavonoids (n = 108) emerged as the dominant subclass, followed by phenolic acids (C6–C1, n = 43), coumarins (n = 31), and phenylpropanoids (C6–C3, n = 20). Classes with fewer than six molecules were grouped as rare subclasses.

A total of 13 compounds appeared in four or more publications, demonstrating broad occurrence across the genus (Figure 2b). Quercetin emerged as the most widely distributed compound, reported in seven publications across five species (*P. odoratissimum*, *P. hortorum*, *P. graveolens*, *P. hispidum*, and *P. zonale*), indicating its fundamental role in *Pelargonium* biochemistry. Rutin and catechin showed similar broad distribution patterns, each appearing in six studies across multiple species.

**Figure 3:**
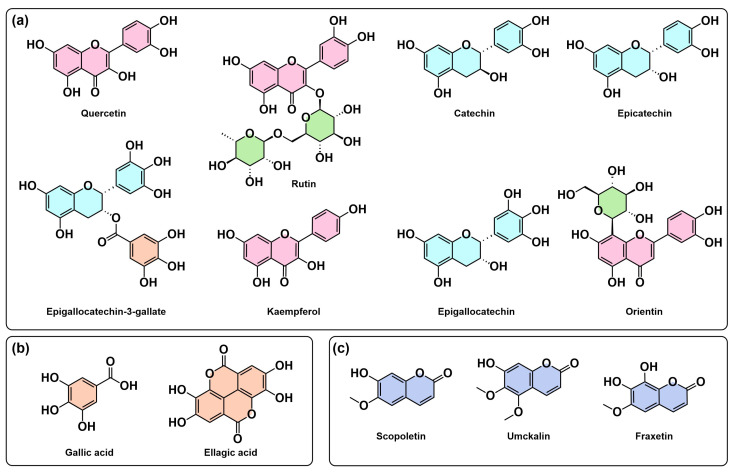
Most-reported molecules in *Pelargonium* species by superclass: (**a**) flavonoids, (**b**) phenolic acids, and (**c**) coumarins.

Chemical diversity analysis revealed both ubiquitous and species-specific compounds (Figure 3). While quercetin and rutin (Figure 3a) demonstrated pan-generic distribution, suggesting conserved biosynthetic pathways, gallic acid (Figure 3b) also appeared frequently across several species. In contrast, compounds like umckalin (Figure 3c) showed restriction to *P. sidoides*, indicating species-specific metabolic specialization. This pattern suggests that while core flavonoid pathways are conserved across *Pelargonium*, secondary modifications and specialized metabolites contribute to species-specific chemical fingerprints with potential taxonomic and pharmacological significance.

### 2.4. Biological Activities from Literature Analysis

**Figure 4:**
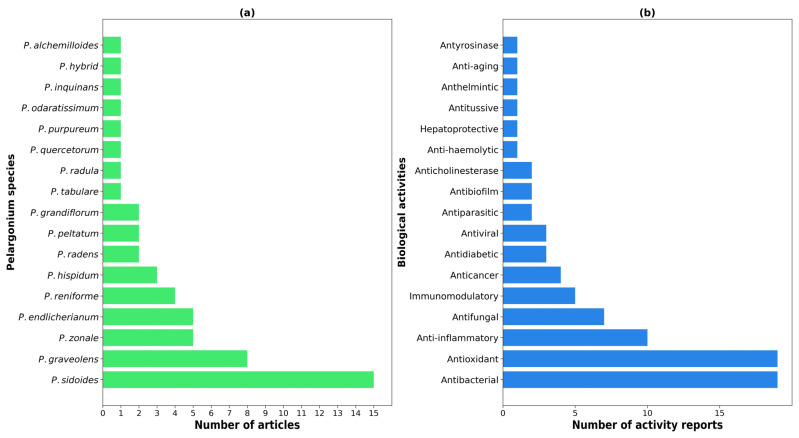
(**a**) Number of studies per *Pelargonium* species reporting biological activity. (**b**) Frequency of each biological activity category identified in *Pelargonium* species.

Literature analysis revealed a broad spectrum of therapeutic effects associated with *Pelargonium* species, with antibacterial, antioxidant, and anti-inflammatory activities predominating (Figure 4a). These effects have been consistently identified across aqueous, alcoholic, and hydroalcoholic extracts, highlighting the phytochemical richness and pharmacological versatility of the genus.

Despite the genus comprising over 280 species, biological activity studies encompassed only 17 different species, representing substantial underexploration (Figure 4b). *P. sidoides* emerged as the most extensively investigated species, with activities reported in 15 publications, including studies of the standardized herbal preparation EPs^®^ 7630 (11% *w*/*w* ethanol extract from *P. sidoides* roots). *P. graveolens* followed with eight reports, while *P. zonale* and *P. endlicherianum* each appeared in five studies and *P. reniforme* in four studies.

Table 1 summarizes the documented biological effects and traditional uses for each *Pelargonium* species. *P. sidoides* and *P. endlicherianum* exhibit the broadest activity profiles, each demonstrating eight distinct therapeutic effects. These pharmacological activities are not only diverse but also highly aligned with their traditional use in folk medicine, particularly for the treatment of respiratory and gastrointestinal infections, wound healing, fever reduction, and immune support.

Antibacterial, antioxidant, and anti-inflammatory activities represent the most commonly reported effects across species. Despite these consistencies, the table also reveals critical knowledge gaps. Several species (e.g., *P. radens*, *P. hispidum*, *P. hybrid*, and *P. tabulare*) exhibit promising biological activities but lack documented uses in traditional medicine, suggesting that their therapeutic potential may be underexplored or culturally underreported. Conversely, a few species such as *P. grandiflorum* and *P. alchemilloides* have known folk applications (e.g., for pneumonia or wound healing) that are supported by preliminary pharmacological evidence but require further experimental validation.

Because an extensive analysis is required to present and discuss biological activities, we included a detailed description and analysis in Supplementary Materials 2 while presenting summarized information in this section. Three main biological activities were the focus of our revision: (1) antimicrobial, (2) antioxidant, and (3) anti-inflammatory effects.

In terms of antimicrobial effects, the main microorganisms across the different publications were *Staphylococcus aureus* (resistant, normal, and clinical strains), *Mycobacterium tuberculosis*, *Escherichia coli*, *Streptococcus pneumoniae*, and *Haemophilus influenzae*. The antioxidant effects of *Pelargonium* extracts have been evaluated using well-known assays, including ferric reducing antioxidant power (FRAP), 2,2-Diphenyl-1-(2,4,6-trinitrophenyl) hydrazyl (DPPH), oxygen radical antioxidant capacity (ORAC), and ABTS. However, the heterogeneity in units and methods (e.g., IC50, percentage inhibition, and Trolox equivalents) complicates direct comparisons, emphasizing the need for standardized protocols.

Articles on anti-inflammatory effects report a variety of approaches, but three principal mechanisms can be discerned: (1) reduction of pro-inflammatory cytokine production (e.g., TNF-α and IL-6), (2) inhibition of cyclooxygenase-2 (COX-2) and prostaglandin release, and (3) suppression of inducible nitric oxide synthase (iNOS) and nitric oxide (NO) production. These mechanisms—described in detail in Supplementary Materials 2—highlight the pathways most frequently modulated in *Pelargonium* extracts, offering insight into their anti-inflammatory capacity.

In addition to the three main activities, other relevant properties were identified, including immunomodulatory, antidiabetic, anticancer, and hepatoprotective effects. Several studies have demonstrated that EPs^®^ 7630 extracts possess significant immunomodulatory properties and enhance the function of peripheral blood phagocytes, increasing phagocytosis, oxidative burst, and intracellular clearance of pathogens such as *Candida albicans*, thereby enhancing the innate immune response [28]. Moreover, it has been demonstrated that EPs^®^ 7630 induces the expression of iNOS and several cytokines (TNF-α, IFN-β, IL-1, IL-12, IL-18, and IFN-γ) in infected cells, promoting transcriptional changes, particularly in the presence of infectious agents [29]. These effects indicate that EPs^®^ 7630 also provides antiviral protection, reducing inflammation and accelerating recovery from upper respiratory tract infections [25].

The ethanolic and aqueous extracts of *P. endlicherianum* have demonstrated significant antidiabetic and anticancer activities. The ethanolic extracts demonstrated inhibition of α-amylase and α-glucosidase enzymes, delaying carbohydrate absorption and reducing postprandial hyperglycemia. Regarding anticancer activity, aqueous extracts of the aerial parts of the plant showed low IC50 values in various cancer cell lines, highlighting their inhibitory capacity. Moreover, the ethanolic extracts of the aerial parts exhibited anticancer activity in the DU-145, HeLa, and MDA-MB-231 cell lines. This activity is attributed to the presence of phenolic compounds, such as ellagic acid, quercetin, and vitexin, which have been demonstrated enzyme-inhibitory and anticancer properties [32].

Conversely, the ethanolic extract of *P. graveolens* demonstrated hepatoprotective and antioxidant activity in mice with carbon tetrachloride (CCl4)-induced liver injury. Treatment with *P. graveolens* for six weeks resulted in a significant reduction in the levels of liver enzymes, alanine aminotransferase (ALT), aspartate aminotransferase (AST), and alkaline phosphatase (ALP), as well as malondialdehyde (MDA). Additionally, it led to an increase in the activity of antioxidant enzymes and the level of reduced glutathione. *P. graveolens* demonstrated comparable hepatoprotective and antioxidant effects to silymarin, as evidenced by improvements in cellular infiltration and hepatic degeneration. The hepatoprotective and antioxidant effects are attributed to its bioactive compounds, primarily flavonoids, flavan-3-ols, and prodelphinidins. These findings indicate that *P. graveolens* may be a promising dietary supplement for the protection of the liver against oxidative stress and liver diseases [41].

Altogether, the biological activities reviewed here support the pharmacological relevance of both well-studied and underexplored *Pelargonium* species. Their broad-spectrum antimicrobial and antioxidant effects, coupled with mechanisms involving immunomodulation and anti-inflammatory action, position the genus as a promising source of therapeutic agents. Further studies employing standardized extraction methods and biological assays are warranted to fully unlock this potential.

### 2.5. In Silico Analysis

To complement the literature-based evidence of biological activities, an in silico analysis was conducted to explore the implications of the identified chemical space. Since the predictive models used did not distinguish between optical isomers, the total number of SMILES employed was reduced. From the 219 chemical structures initially classified as “0,” 197 unique molecules remained after removing duplicates based on stereochemistry.

**Figure 5:**
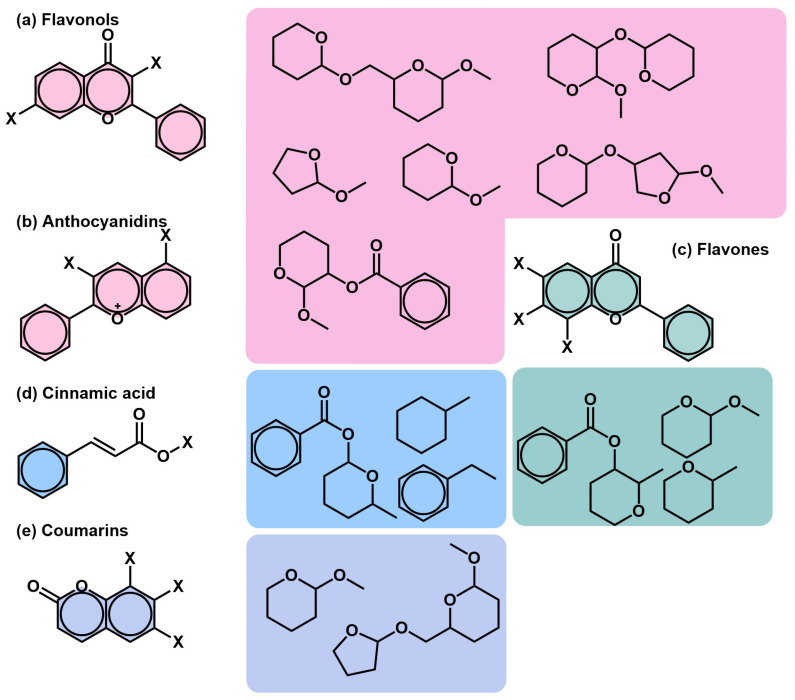
Representative scaffolds and common structural derivatives in *Pelargonium* extracts. (**a**) Flavonols. (**b**) Anthocyanidins. (**c**) Flavones. (**d**) Cinnamic. (**e**) Coumarins. Colored boxes to the right illustrate frequently observed substitution patterns or glycosylated forms corresponding to each class.

The main scaffolds representing the most common derivatives are shown in Figure 5, with the exception of the gallotannins. The full list of 219 SMILES with their chemical classification can be found in Table A in Supplementary Materials 1.

The largest class, referred to as C3: Glycosylated flavonols, comprises 26 molecules (Figure 5a). These compounds share a common flavonol scaffold with several possible modifications, including 3-O and 7-O glycosylations. The glycosylations involve one or more sugar moieties, which may themselves have derivations such as methylations or carboxylic groups. The second-largest class, C1: Glycosylated gallotannins, contains 21 molecules characterized by their complex scaffolds and extensive modifications. Another class with 21 molecules is C4: Non-glycosylated coumarins (Figure 5e), which lack glycosylations but exhibit diverse structural modifications.

A smaller class, C15: Glycosylated anthocyanidins, includes 10 molecules (Figure 5b) with numerous modifications related to the type and number of glycosylations, as well as derivative sugar moieties. Similarly, C2: Flavones, comprising 10 molecules, are typically glycosylated or contain other derivatives, often located at the C-6 position (Figure 5c). C20: Cinnamic acids and derivatives, also with 10 molecules (Figure 5d), include both simple and modified forms. C11: Simple phenolic acids, a class of nine molecules, are defined by the absence of glycosylations. Finally, C7: Glycosylated coumarins, the smallest of these prominent classes with six molecules, consists of coumarin structures modified by glycosylation. 

**Figure 6:**
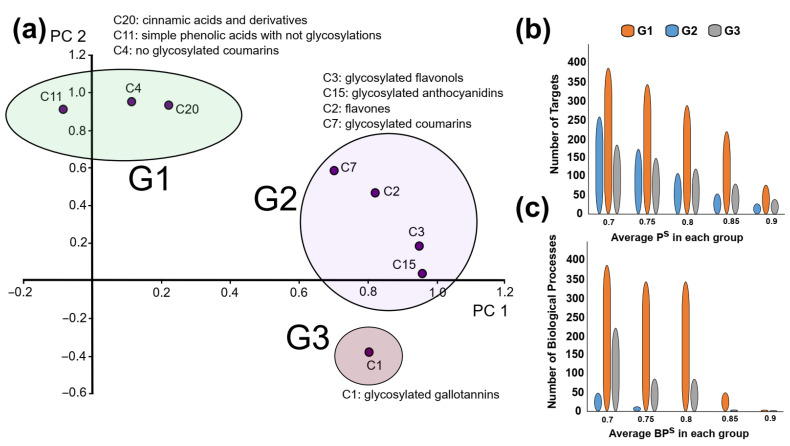
(**a**) PC1 vs. PC2 component representation of each of the chemical classes. (**b**) Number of targets with average $P_{i,j}^{S}$ higher than the defined cutoff. (**c**) Number of biological processes with ${BP}_{i,j}^{S}$ each group higher than the defined cutoff.

To explore the relationships between chemical classes and their biological targets, the protein score ($P_{i,j}^{S}$) was computed for 526 proteins to evaluate compound–target interactions. Detailed protein scores for all classes are provided in Table B in Supplementary Materials 1, while classifications by clusters and groups are available in Tables C and D in Supplementary Materials 1, respectively. A principal component analysis (PCA) was performed, revealing two major components with a cumulative explained variance of 92.02%. The PC1 vs. PC2 plot (Figure 6a) identified three main molecular clusters with distinct protein interaction profiles. The G2 cluster, comprising flavonoids and glycosylated coumarins, contained the largest number of proteins with high $P_{i,j}^{S}\geq 0.8$.

Interestingly, the predicted protein interaction profile for glycosylated coumarins was more like flavonoids than that of non-glycosylated coumarins. This similarity is highlighted in Figure 6b, where G2 proteins exhibit a higher concentration of significant interactions compared to other groups. Restricting the analysis to proteins with $P_{i,j}^{S}\geq 0.8$ revealed 19 proteins that are shared among the three groups. These proteins include growth hormone secretagogue receptor type 1 (GHSR), sodium-dependent serotonin transporter (SC6A2), RAC-gamma serine/threonine-protein kinase (AKT3), C-X-C chemokine receptor type 4 (CXCR4), cholecystokinin receptor type A (CCKAR), proteasome subunit beta type-1 (PSB1), lysine-specific demethylase 4A (KDM4A), transcription factor p65 (TF65), ephrin type-A receptor 2 (EPHA2), catenin beta 1 (CTNB1), glycogen synthase kinase-3 alpha (GSK3A), protein kinase C beta type (KPCB), protein kinase C epsilon (KPCE), tachykinin receptor 1 (NK1R), phosphatidylinositol 4-kinase beta (PI4KB), ribosomal protein S6 kinase alpha-3 (KS6A3), glutamate carboxypeptidase 2 (FOLH1), cathepsin L2 (CATL2), and C3a anaphylatoxin chemotactic receptor (C3AR). These shared proteins are associated with pathways relevant to inflammation, metabolic regulation, and cellular signaling, highlighting their potential biological significance.

The biological processes associated with these molecular groups were analyzed using the biological process score e ${BP}_{i,j}^{S}$. The results revealed that G2 and G3 had the highest number of significant biological processes ${BP}_{i,j}^{S}>0.8$, while no significant biological processes were identified for G1 (non-glycosylated coumarins and phenolic acids). This is illustrated in Figure 6c. A detailed list of all biological processes and their associated scores is provided in Table E, while Table F presents all biological processes by group in, both in Supplementary Materials 1.

The molecular groups within G2 were found to have a more significant impact on biological processes compared to other groups. This is reflected in the high number of biological processes associated with G2, particularly those with ${BP}^{G2}>0.85$. To handle the extensive dataset, semantic simplification was performed using Revigo. The complete set of simplified biological processes is provided in Table G in Supplementary Materials 1, while the most relevant processes for G2 are summarized in Table 2. These processes highlight the functional diversity of G2 molecules and their potential involvement in key biological activities.

Table 2 highlights the strong relationship between G2 molecules and several inflammation-related biological processes. The application of Revigo revealed that response to dexamethasone (GO:0071548) and cellular response to prostaglandin E stimulus (GO:0071380) are central processes associated with G2. These processes are encompassed within the long-chain fatty acid biosynthetic process (GO:0042759), which includes arachidonic acid secretion (${BP}^{G2}=0.81$), arachidonic acid metabolic process (${BP}^{G2}=0.81$), prostaglandin biosynthetic process (${BP}^{G2}=0.80$), positive regulation of prostaglandin secretion (${BP}^{G2}=0.76$), and prostaglandin metabolic process (${BP}^{G2}=0.71$). These findings underscore the significant role of G2 molecules in modulating prostaglandin-related pathways, which are critical for inflammation regulation.

In addition, other biological processes may be associated with other biological activities, such as the G protein-coupled acetylcholine receptor signaling pathway, which is linked to insulin response and fatty acid metabolism. It is plausible that both are associated with an antidiabetic effect. Furthermore, the involvement of telomerase activity, tachykinin receptors, and even bradykinin may be associated with anticancer effects.

## 3. Discussion

Although the genus *Pelargonium* comprises more than 280 recognized species, it remains significantly underexplored regarding its phytochemical and pharmacological potential. Our systematic review of 176 studies revealed that only 10 species have undergone comprehensive chemical characterization, representing less than 4% of the genus. Consequently, over 96% of *Pelargonium* species remain uncharacterized, highlighting a substantial knowledge gap that parallels trends observed in other medicinally important plant genera [79].

Strikingly, despite the African origin and traditional use of *Pelargonium*, only Egypt and South Africa have contributed studies to its chemical characterization. Egyptian research has primarily focused on synthesizing nanoparticles from aqueous extracts of *P. odoratissimum* and evaluating their antioxidant and antibacterial properties [80]. Other studies have explored the antiviral activity of *P. zonale* against human coronavirus 229E [81] and the hepatoprotective effects of *P. graveolens* ethanolic extracts [41]. In South Africa, the research aimed to identify the anthocyanin profile in the flowers of *P. zonale*, *P. grandiflorum*, and *P. hortorum*, evaluating their potential as natural food colorants [82].

These few studies, however, lack comprehensive secondary metabolite profiling or the structural elucidation of novel compounds. This geographic and scientific bias restricts our understanding of the genus and limits its potential applications in both traditional and modern medicine, especially given its rich ethnobotanical use in southern Africa for treating respiratory, gastrointestinal, and dermatological conditions.

To address this imbalance and promote a more inclusive research landscape, future investigations should consider fostering collaborations with African research institutions, particularly those with expertise in ethnomedicine and biodiversity conservation. Encouraging the development of regional and international networks could help bridge current collaboration gaps, promote equitable scientific exchange, and generate contextually relevant and scientifically robust outputs. These efforts would facilitate deeper phytochemical exploration of *Pelargonium* species in their native range and contribute to unlocking their full pharmacological potential.

In this review, we focused on aqueous, alcoholic, and hydroalcoholic extracts excluding essential oils due to their reliance on apolar solvents. Even within these constraints, minor quantities of fatty acids and terpenoids were detected. Predominant classes identified included flavonoids, phenols, coumarins, and phenylpropanoids, consistent with the extraction methods employed.

Three primary analytical techniques were commonly employed in the reviewed literature: HPLC-UV/DAD, LC-MS, and NMR, either individually or in combination. HPLC-UV or HPLC-DAD are accessible and widely used for comparing retention times and UV spectra to known standards. However, their reliance on these standards inherently limits their ability to identify novel or uncommon compounds. This methodology was employed in the investigation of four species: *P. zonale*, *P. odoratissimum*, *P. quercetorum*, and *P. hispidum*. In the latter two species, this technique was the sole method employed for their characterization, which underscores the necessity to use techniques more related to untargeted metabolomics analysis to increase molecular characterization. This is particularly pertinent given that in the study by Williams et al. [83] on the phenolic profile of the genus *Pelargonium*, the presence of ellagitannins was identified in *P. quercetorum*, yet there is no description of *P. hispidum*.

In nine additional studies, HPLC-MS was employed, techniques that are more powerful in terms of compound separation and identification. However, it should be noted that this methodology also has limitations, as its effectiveness is contingent upon the availability of databases for compound identification. Finally, in publications focused on *P. sidoides* and *P. reniforme*, where highly oxidized coumarins, O-galloyl-C-glycosyl flavones, pelargoniins, and ellagitannins were described, a combination of HPLC-MS and NMR was employed. These techniques facilitate the identification of new compounds, as observed throughout the review of these two species.

The application of any of these techniques, as well as the type of data analysis (especially in NMR and MS), has a profound impact on the chemical diversity that can be identified. The challenges inherent to metabolomic research, particularly those about spectrum annotation [84,85] and the diversity of data analysis and experimental procedures, have a significant impact on the number of identified compounds and the discovery of potential new ones. For example, of the nine articles that employed some form of liquid chromatography combined with mass spectrometry (LC/HPLC/UPLC-MS), 245 compounds were identified, of which 167 were unique after the removal of duplicates. Similarly, of the five studies that employed a combination of HPLC-MS and NMR, 80 compound annotations were recorded, with 54 being unique once repetitions were excluded.

Despite these efforts, targeted metabolomics still dominates, limiting the detection of unexpected metabolites. The use of untargeted metabolomics, in tandem with chemoinformatic tools, could enable broader chemical space mapping and accelerate novel compound discovery.

Many of the studies focused on *Pelargonium*’s biological activities are limited by their generalized chemical composition analyses, often restricted to total polyphenols, flavonoids, or other broad categories. Of the 176 reviewed articles, only 17 species have been studied for their biological activities, further underscoring the restricted scope of research in this area. This limitation is compounded by variability in experimental assays, particularly those used to evaluate antioxidant activity, such as DPPH, ABTS, and FRAP. These methodological inconsistencies hinder direct comparisons across studies and prevent meaningful meta-analyses. Similarly, antibacterial studies often target different organisms using diverse methodologies, further complicating cross-study evaluations.

These limitations underscore the critical need for methodological standardization in antioxidant and antimicrobial assays, which represents a major obstacle to reproducibility and cross-study comparability in natural products research. In antioxidant evaluations, differences in methodologies, result units, concentration ranges, and endpoint criteria severely limit meaningful comparisons. Similarly, antimicrobial studies often vary in strain selection, culture conditions, inoculum preparation, and susceptibility protocols, hindering the development of reliable efficacy profiles.

To address these challenges, the adoption of internationally recognized protocols is essential. For antimicrobial testing, either the Clinical and Laboratory Standards Institute (CLSI) [86] or European Committee on Antimicrobial Susceptibility Testing (EUCAST) [87] guidelines offer standardized procedures for strain selection, media composition, inoculum density, and result interpretation. For antioxidant assays, AOAC Official Methods provide harmonized standards for sample preparation, reagent concentrations, and result expression (preferably in Trolox equivalents) to support cross-study integration [88].

Systematic implementation of these validated frameworks is essential to strengthen the scientific foundation of natural product research, enabling the generation of high-quality, reproducible data that can inform evidence-based therapeutic development. Additionally, computational approaches employed in natural product research face inherent methodological limitations that must be acknowledged. One important methodological limitation of our computational analysis is that the target-centric models used do not distinguish between stereoisomers since they are based on 2D molecular fingerprints and descriptors that do not encode stereochemistry. This is particularly relevant given that stereochemistry plays a crucial role in molecular recognition and biological activity, especially in natural products like flavonoids. As highlighted by Scott et al. [89], more than 60% of FDA-approved drugs contain at least one stereocenter, and many exhibit stereoselective binding to their targets. Therefore, the exclusion of stereochemical information may underestimate or misrepresent certain compound–target interactions. Future studies should consider integrating stereospecific modeling tools or experimental validation to overcome this limitation.

*P. sidoides* has garnered significant attention due to its role in producing the phytopharmaceutical EPs^®^ 7630, which is widely used to treat respiratory tract infections such as acute bronchitis, asthma, and tuberculosis [31,90,91,92]. The anti-inflammatory properties of EPs^®^ 7630 have been demonstrated through inhibition of lipopolysaccharide-induced responses [93].

Furthermore, it has shown promise in treating diarrhea [91]. The therapeutic potential of EPs^®^ 7630 is primarily attributed to its cytoprotective and antibacterial properties [94]. The phytopharmaceutical EPs^®^ 7630 is characterized by its high concentration of oxygenated coumarins, primarily umckalin and other sulfated coumarins. Given that several articles include this phytopharmaceutical preparation, it is expected to have some coumarins in the group of molecules commonly detected.

The differences in the chemical profiles of roots and leaves are likely significant, but a lack of comparable data limits further discussion. Consistent therapeutic themes include antibacterial, anti-inflammatory, and antioxidant effects, with some studies also reporting antidiabetic, antifungal, and anticancer activities.

The analysis of the chemical space using compound–target predictive models suggests that, in terms of target profiles, the main responsibility of biological functions is located in the flavonoids group, especially glycosylated flavonoids (e.g., rutin and quercetin 3-o-glucosyde), glycosylated anthocyanidins (e.g., glycosylated petunidin and cyanidin), flavones (e.g., luteolin and glycosylated luteolin), and glycosylated coumarins (instead of non-glycosylated coumarins, e.g., xeroboside and aesculin). As previously explained, it is difficult to compare the chemical space across distinct species. Accordingly, we have two principal functional analyses: one focused on the overall target profile and the other based on chemical groups. In both instances, target-specific data and biological processes are significant.

The biological processes presented reflect some of the known biological effects explored in the literature review. The implications of arachidonic acid and prostaglandin release have been identified. Based on the identified targets, it is consistent with the presence of polyunsaturated fatty acid lipoxygenase (ALOX15 and score of 0.87), prostaglandin E2 receptor EP4 subtype (PTGER4 and score of 0.88) in the top 30 of all proteins, and polyunsaturated fatty acid 5-lipoxygenase (ALOX5 and score of 0.76 in G2). Another possible target is prostaglandin E synthase (PTGES with a score of 0.88 specifically in G2). The three groups are involved in these processes, but particularly groups 2 and 3 (flavonoids and gallotannins), which exhibited the highest scores. Regarding PTGER4, the G1 also exhibits a high score, indicating the potential for effects of phenolic acids. Several experimental studies support our findings. The inhibitory effect of quercetin, kaempferol, and other flavonoids on 15-lipoxygenase (ALOX15) is well-attested in enzymatic assays [95,96]. These flavonoids are highly represented in *Pelargonium* species.

The effect of flavonoids on nitric oxide metabolism has been extensively explored. In general terms, we have several possible mechanisms [94]: (1) based on oxidant and antioxidant capacity of flavonoids (basically reaction with NO, O2-, etc.) and (2) regulation of NOS1, NOS2, or NOS3. The first approach is based on chemical characteristics and is not properly related to a target-based model (as used in this work). In the second approach, there is repeated evidence of the NOS2 expression reduction associated with molecules like quercetin, kaempferol, and apigenin, as well as some of their glycosylated derivation [94,97]. In our results, the NOS2 is predicted as a potential target with an average score of 0.82 for both G1 and G2. Other works also found a good theoretical binding affinity of several flavonoids to NOS2 [98]. However, the inhibition of NOS2 activity by flavonoids is controversial in experimental terms. Some previous works did not find any interaction [97], while other authors report inhibition activities in a wide range of IC50 values [94,95,99] for specific groups of flavonoids (e.g., some flavanols, genistein, chrysin, quercetin, rutin, kaempferol, etc.). Additionally, in the top 30 protein targets with higher scores, we have the nuclear factor kappa-light-chain-enhancer of activated B cells (NF-κB) p65, which is not only related to inflammation [100] but also affected by flavonoids like kaempferol, which had been identified in many of the species in our study [101]. These discrepancies highlight the importance of rigorous experimental validation using standardized protocols to confirm computational predictions and resolve existing controversies in literature.

Building on the identified inflammatory targets (ALOX15, PTGER4, ALOX5, and PTGES), experimental evidence supports these computational predictions through established mechanisms of structurally related compounds. Interestingly, these predicted inflammatory targets align with established mechanisms of structurally related isocoumarin compounds, which have demonstrated dual inhibitory activity against both ALOX5 and PGE2 pathways [102,103]. Recent studies have shown that 3-aryl isocoumarin derivatives act as potent anti-inflammatory agents through redox-type mechanisms, with compounds containing dihydroxyl groups exhibiting superior antioxidant activity. This multi-target approach, simultaneously affecting lipoxygenase and prostaglandin synthesis pathways, may provide enhanced therapeutic efficacy compared to single-target inhibition strategies [102,103]. Given the structural similarities between coumarins found in *Pelargonium* species and these bioactive isocoumarin scaffolds, similar mechanisms may contribute to the observed anti-inflammatory effects.

Interestingly, our PCA-based clustering revealed that glycosylated coumarins (G2) clustered more closely with flavonoids than with their non-glycosylated analogs (G1), suggesting that glycosylation exerts a stronger influence on the compound–target interaction profile than the core scaffold itself. This supports the notion that sugar moieties can significantly modulate a molecule’s physicochemical and pharmacokinetic properties—such as solubility, membrane permeability, or binding affinity—thereby redefining its biological behavior [104]. This finding also suggests that, at least in this group, glycosylation may play a more dominant role in determining a compound’s target interaction profile than its core scaffold itself. This challenges traditional structure–activity relationship paradigms that primarily focus on the core molecular framework. Moreover, it points toward the benefits of combining biological profile clustering with traditional chemical similarity. The sugar moiety impact on inflammatory response (and other biological activities) has been documented in flavonoids [105] and in coumarins [106]. Nevertheless, as far as we know, no previous study had been performed comparing both chemical groups and their structure–activity relationships. Further computational and experimental research should be needed in this direction.

Conversely, translating these promising computational findings to clinical applications requires careful consideration of pharmacokinetic challenges, particularly for the glycosylated compounds identified as key bioactive molecules. Glycosylated flavonoids typically exhibit poor oral absorption due to their hydrophilic nature and dependence on specific transporters and enzymes for intestinal uptake. The absorption of flavonoid glycosides is largely determined by the sugar moiety, with only certain glycosides (particularly glucosides) being substrates for lactase–phlorizin hydrolase (LPH) and sodium-dependent glucose transporter 1 (SGLT1) in the small intestine [107,108,109]. Glycosides that are not substrates for these enzymes must reach the colon for bacterial hydrolysis, resulting in significantly lower bioavailability compared to their aglycone counterparts [108].

However, recent evidence suggests that glycosylated flavonoids may exert therapeutic effects through alternative mechanisms, including local gastrointestinal action and potential deglycosylation by intestinal enzymes to release more bioavailable aglycones [110,111]. For chronic disease management, these pharmacokinetic limitations highlight the need for innovative formulation strategies, such as nanoencapsulation, liposomal delivery systems, or co-administration with absorption enhancers, to improve bioavailability and therapeutic efficacy [112,113].

Interestingly, our in silico analysis also points toward other possible targets and mechanisms related to other diseases, for instance, the relationship with G protein-coupled acetylcholine receptor signaling pathway, which is directly involved with insulin responses. Moreover, the second target with the highest score is the glycogen synthase kinase-3 alpha (GSK3A) (global score of 0.93). The inhibition of GSK3A improves insulin response [114], suggesting possible beneficial effects on insulin resistance, metabolic syndrome, and diabetes [115]. Moreover, the target with the highest global score, which is probably affected by several plant metabolites, is the phosphatidylinositol 4-kinase beta (PI4KB). In recent studies, this protein has also been related to glucose transporter type 4 (GLUT4) translocation and consequently points toward an interesting target for diabetes and insulin resistance therapy [116].

Some experimental studies support our in silico findings, particularly for ALOX15 and PI4KB. Although the biological roles of the targets are well established, most of the supporting studies rely on in vitro enzymatic assays. In contrast, reports demonstrating functional inhibition in cellular or animal models remain relatively less common. For instance, Liu et al. [117] showed that scutellarein inhibits ALOX15 activity and reduces ferroptosis in lung epithelial cells and a mouse model, using techniques such as localized surface plasmon resonance (LSPR), drug affinity responsive target stability (DARTS), and the cellular thermal shift assay (CETSA). Similarly, 8-farnesyloxycoumarin decreased the viability of prostate cancer cells overexpressing ALOX15 [118], and delphinidin (identified in *Pelargonium*) was shown to reduce ferroptosis and myocardial damage by suppressing ALOX15 expression both in vitro and in vivo [119]. In the case of PI4KB, Arita et al. demonstrated that pachypodol inhibits poliovirus replication by disrupting PI4KB-mediated PI4P synthesis [120]. Although these compounds, with the exception of delphinidin, are not derived from *Pelargonium* species, their functional relevance highlights our confidence in the relevance of the predicted targets, as they can indeed be modulated by phytochemicals from other plant sources. These findings support the biological importance of ALOX15 and PI4KB and underscore the need to experimentally validate whether *Pelargonium*-derived metabolites exhibit similar activities, ideally through integrated approaches combining target engagement assays with functional cellular or animal studies.

There are numerous additional intriguing targets and associated mechanisms that warrant experimental investigation and validation (e.g., aldosterone synthase, growth hormone secretagogue receptor type 1, etc.). However, there is a need to shift the focus from global extract analysis to individual compound interactions. Such studies will not only elucidate the chemical composition but also identify specific molecules, thereby narrowing the potential pharmacological actions. Future studies should consider integrating stereospecific modeling tools or experimental validation to overcome computational limitations.

## 4. Materials and Methods

### 4.1. Literature Search for Chemical Composition Studies

To identify relevant studies on the metabolite profiles of *Pelargonium* extracts, a comprehensive search was conducted in the Scopus database. The search terms included TITLE-ABS-KEY (*Pelargonium* AND extracts) AND TITLE-ABS-KEY (methanol OR methanolic OR ethanol OR ethanolic OR alcohol OR alcoholic OR hydroalcoholic OR water OR aqueous OR metabolite OR profile OR nanoparticles) AND NOT TITLE-ABS-KEY (essential AND oils OR volatile AND oils OR oils). The search was restricted to articles published between 1999 and 2025 and limited to English-language publications. The term “nanoparticles” was included to capture studies employing advanced characterization techniques, although studies focused exclusively on nanoparticle synthesis were subsequently excluded during the screening process.

**Figure 7:**
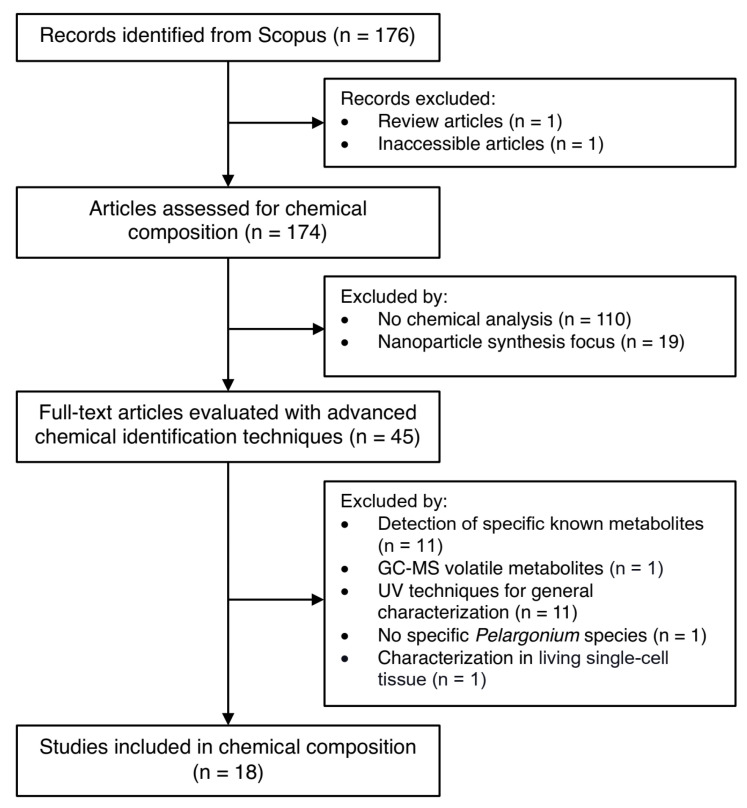
Schematic overview of included and excluded manuscripts for chemical composition in *Pelargonium* species.

The initial search retrieved 176 articles, which underwent manual screening and evaluation. Studies were included if they provided comprehensive chemical characterization using advanced analytical techniques, including ultraviolet (UV) spectroscopy, high-performance liquid chromatography (HPLC), mass spectrometry (MS), or nuclear magnetic resonance (NMR). These analytical methods were selected for their demonstrated reliability in detailed metabolite profiling. The complete selection process is illustrated in Figure 7.

After applying the inclusion and exclusion criteria, 18 studies met the requirements for detailed analysis. These studies employed diverse extraction methods, including water-based extractions (infusions and decoctions), acidified water, alcoholic solutions (methanol and ethanol), and hydroalcoholic mixtures with varying water-to-alcohol ratios. Additionally, five studies utilized acetone–water combinations, though only the aqueous fractions were analyzed for chemical composition.

### 4.2. Literature Search for Biological Activity Studies

A parallel search strategy was implemented to identify studies examining the biological activities of *Pelargonium* extracts. Using the same database and temporal restrictions as described in Section 4.1, the following search terms were employed: TITLE-ABS-KEY (*Pelargonium* AND extract AND activity) AND TITLE-ABS-KEY (methanolic OR methanol OR ethanolic OR ethanol OR alcoholic OR hydroalcoholic OR water OR aqueous), while excluding studies on essential oils or nanoparticles.

**Figure 8:**
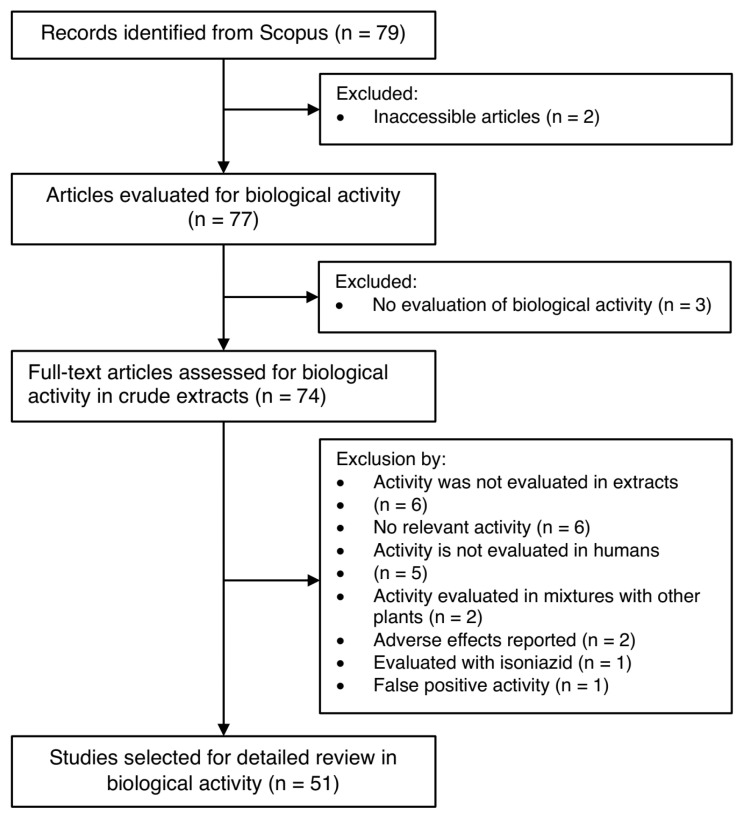
Schematic overview of included and excluded manuscripts for biological activity in *Pelargonium* species.

This search yielded 79 articles, which underwent systematic screening through evaluation of titles, abstracts, methodologies, and reported biological activities. The selection process is detailed in Figure 8. Studies were excluded if they (i) did not focus on biological activity of crude extracts, (ii) evaluated essential or volatile oils, (iii) included plant mixtures rather than pure *Pelargonium* extracts, (iv) reported only adverse effects, or (v) investigated synergistic effects with synthetic drugs (e.g., isoniazid). This final criterion ensured that observed biological activities could be attributed solely to *Pelargonium* extracts without confounding pharmacological influences.

Following application of these criteria, 51 studies were included in the final analysis, providing a comprehensive foundation for evaluating the biological potential of various *Pelargonium* species in crude extract forms.

### 4.3. Chemical Data Curation and Molecular Classification

Chemical nomenclature standardization was performed to address the significant ambiguity in molecular identification reported across different studies, as previously described by our group [79]. Manual curation was conducted using PubChem (https://pubchem.ncbi.nlm.nih.gov/, accessed on 7 January 2025) and J-GLOBAL (https://jglobal.jst.go.jp/en, accessed on 9 January 2025) databases, selected for their comprehensive coverage and reliable annotations of natural products.

A three-tier classification system was implemented based on structural certainty:

Label “0” was assigned to molecules with unambiguous identification, where names were directly matched in PubChem or J-GLOBAL databases or were explicitly described with structural representations in source publications. For these compounds, SMILES (Simplified Molecular Input Line Entry System) codes were retrieved, and InChIKey (IUPAC International Chemical Identifier) codes were generated using RDKit library in Python (version 3.13).

Label “1” was assigned to molecules not found in the databases, such as kaempferol–pentose, kaempferol–hexose–rhamnose, or prodelphinidin dimer, which lacked a corresponding SMILES code or detailed chemical structure.

Label “2” was designated for molecules with high structural uncertainty, including those described as “isomers” or “derivatives” (e.g., “hexahydroxydiphenic acid isomer” and “Saccharumoside C hexoside derivative”). For these compounds, the closest available structural analogs were selected to maintain chemical space diversity.

All compounds were further classified using NPClassifier; the molecules were classified using their SMILES representation and the NPClassifier [121], a deep neural network that categorizes natural products into pathway, superclass, and class based on SMILES input. This allowed grouping into biosynthetically relevant subclasses such as glycosylated flavonols, phenolic acids, and coumarins.

### 4.4. Compounds–Target Protein Interaction Prediction and Analysis

To investigate possible mechanisms of action, we adopted a methodology similar to that described by Beltrán-Noboa et al. [122]. Compound–target predictions were performed using target-centric models (TCM) [123], which are ligand-based models constructed for each individual target using machine learning algorithms trained on bioactivity data from the ChEMBL database. Specifically, the TCMs were implemented [123] using a consensus strategy of 15 individual models. These models combine several artificial intelligence algorithms (support vector machine, random forest classifiers, decision trees, and others) using molecular fingerprints and physicochemical descriptors to capture structure–activity relationships. These models achieve an average predictive accuracy above 80% across 526 protein targets. The TCMs do not differentiate stereoisomers, which is a known limitation and should be considered when interpreting the results [123].

The NPClassifier classification of SMILES was expanded to include information about glycosides attached to the ring or main structure. Previous studies [124,125] have shown significant bioactivity differences between glycosylated and non-glycosylated structures. Incorporating glycoside data facilitated the formation of analytical clusters and clarified relationships between structural features and biological outcomes. Clusters with fewer than five compounds were excluded to ensure statistical robustness.

The protein score ($P_{i,j}^{S}$) for a protein “*i*,” in cluster “*j*,” was computed as shown in Equation (1):

(1) $$P_{i,j}^{S}=\sqrt{\frac{\langle I_{i,j}^{S}\rangle \cdot F_{j}}{N_{j}}}$$

where $\langle I_{i,j}^{S}\rangle$ is the average of all predicted compound–protein interaction scores (for protein “*i*”), excluding 0 values, for all smiles in the cluster “*j*”; $N_{j}$ is the number of smiles in the cluster “*j*”; and $F_{j}$ is the number of smiles in the cluster “*j*” with a score higher than 0.

The score ranges between 0 and 1, with higher values indicating a greater likelihood of interaction. A principal component analysis (PCA) with varimax rotation was performed to visualize clustering among chemical classes and their predicted interaction profiles. The PCA captured the variance in interaction patterns and helped delineate structure–activity relationships.

### 4.5. Functional Annotation of Targets and Biological Process Analysis

To translate predicted protein targets into biologically meaningful insights, functional annotation analysis was performed using the DAVID Bioinformatics Resources tool [126] to identify associated biological processes. The objective was not enrichment per se, as this depends on the protein space of the predictive models, but rather to implement a weighting procedure. For each biological process, a weighted score (${BP}_{i,j}^{S}$) was calculated for each cluster by averaging the $P_{i,j}^{S}$ scores of all proteins involved in the process. This served as a metric for the impact of each chemical cluster on specific metabolic pathways.

To simplify and refine the analysis of biological processes, we used Revigo [127], which reduces redundancy in Gene Ontology (GO) term lists by applying semantic similarity measures. Revigo also evaluates term frequency, uniqueness, and dispensability, enabling the identification of the most relevant and distinctive biological processes.

## 5. Conclusions

This comprehensive review reveals substantial underexploration of the *Pelargonium* genus despite its remarkable diversity and extensive traditional medicinal use. From over 280 recognized species, only 10 have undergone detailed chemical characterization, and merely 17 have been evaluated for biological activities, representing a critical knowledge gap that limits both scientific understanding and therapeutic development.

Strikingly, this underexploration is compounded by significant geographic research bias, with only Egypt and South Africa contributing to chemical characterization studies, despite the genus’s African origin and rich ethnobotanical heritage across the continent.

Our analysis identified glycosylated flavonoids as the predominant bioactive chemical class in *Pelargonium* species, with computational predictions indicating their primary role in modulating inflammation, oxidative stress, and metabolic pathways. Notably, these findings align remarkably well with traditional medicinal uses for respiratory, gastrointestinal, and dermatological conditions, as exemplified by the successful development of EPs^®^ 7630 from *P. sidoides* for respiratory tract infections.

Key therapeutic targets identified through in silico analysis include inflammatory mediators (ALOX15, ALOX5, PTGES, and NOS2) and metabolic regulators (GSK3A and PI4KB), providing mechanistic insights into both traditional uses and potential novel applications, particularly for chronic inflammatory and metabolic disorders. However, the clinical translation of these promising findings faces significant pharmacokinetic challenges, as glycosylated flavonoids typically exhibit poor oral bioavailability, necessitating innovative formulation strategies, such as nanoencapsulation or liposomal delivery systems. Methodological heterogeneity across studies, particularly in antioxidant and antimicrobial assays, significantly hampers comparative analysis and limits the development of evidence-based therapeutic protocols.

## 6. Future Perspectives

To address the identified limitations and unlock the full therapeutic potential of *Pelargonium* species, several strategic research directions should be prioritized:

Methodological standardization through the adoption of internationally recognized standards such as CLSI [86]/EUCAST [87] guidelines for antimicrobial testing and AOAC methods [88] for antioxidant evaluations is essential to enable meaningful cross-study comparisons and evidence-based therapeutic development.

Systematic chemical profiling of underexplored species using untargeted metabolomics approaches, particularly utilizing LC-HRMS platforms with standardized analytical workflows and comprehensive database integration, is crucial for enhanced compound identification and novel compound discovery. To ensure consistency and comparability across studies, future research should adopt well-defined workflows that include optimized sample preparation protocols, LC-HRMS analysis using both positive and negative ionization modes, feature detection and alignment with tools such as MZmine or XCMS, multivariate statistical analysis for biomarker discovery, and structural annotation through MS/MS spectral databases and molecular networking platforms like GNPS.

Computational predictions require experimental validation through targeted studies, particularly for glycosylated compounds that exhibit unique pharmacokinetic properties. The clustering of glycosylated coumarins with flavonoids rather than their non-glycosylated analogs suggests that sugar moieties fundamentally alter compound–target interaction profiles, challenging traditional scaffold-based classification approaches.

The transition from extract-based investigations to compound-specific mechanistic studies represents a crucial evolution necessary to identify molecules responsible for observed biological activities and support rational drug development.

Addressing pharmacokinetic limitations through innovative formulation strategies such as nanoencapsulation or liposomal delivery systems will be essential for clinical translation of glycosylated flavonoids. Future computational studies should integrate stereospecific modeling tools and experimental validation specifically designed for glycosylated natural products to overcome current limitations and provide more accurate structure–activity relationships.

The successful implementation of these research priorities will enable the rational development of standardized therapeutic agents for managing chronic inflammatory and metabolic diseases while honoring the rich traditional knowledge associated with this diverse genus.

## Figures and Tables

**Table 1:** Biological activities and traditional uses reported in each *Pelargonium* species.

*Pelargonium* Species	Biological Activities	Traditional Uses
*P. sidoides*	Antioxidant [18,19], antimicrobial (including antiviral, antifungal, and antibacterial) [6,18,20,21,22,23,24,25], antibiofilm [18], anti-inflammatory [26,27], immunomodulatory [25,28,29,30], and antitussive [26]	Respiratory infections, gastrointestinal disorders, fever, wound healing, immune support [1,14,25,27,31]
*P. endlicherianum*	Antioxidant [32,33], antibacterial [34], anti-inflammatory [35], antidiabetic, anticholinesterase, anticancer, anti-tyrosinase [32], and anthelmintic [36]	Respiratory infections, menstrual pain, gastrointestinal infections, skin diseases, anthelmintic [32,37]
*P. graveolens*	Antioxidant [38,39,40,41], antibacterial [42,43,44,45], anti-aging [38], and hepatoprotective [41]	Respiratory infections, gastrointestinal disorders, dermatological conditions, gynecological disorders, nervous system disorders [46,47,48]
*P. zonale*	Antioxidant [49,50,51], antibacterial [52], anti-inflammatory [53], and anticancer [51]	Respiratory disorders, gastrointestinal conditions, cardiovascular support, hemostatic effects, wound healing [54,55]
*P. reniforme*	Antioxidant [56,57] and antimicrobial (antibacterial and antifungal) [6,57,58]	Gastrointestinal disorders, respiratory infections, genitourinary conditions [1,14,57,59]
*P. hispidum*	Antioxidant [50], anti-inflammatory [53], and antidiabetic [60]	No specific ethnobotanical information available
*P. peltatum*	Antibacterial [61,62]	Respiratory and oral conditions, dermatological uses, cosmetic applications [61,63]
*P. radens*	Antioxidant, anticancer [51], and anti-inflammatory [53]	No specific ethnobotanical information available
*P. odoratissimum*	Antiparasitic [64] and anticancer [65]	Cardiac stimulant, cold and inflammation treatment, air disinfection, respiratory tract disorders [66,67,68]
*P. grandiflorum*	Anti-inflammatory [53] and antidiabetic [60]	Pneumonia treatment [69]
*P. alchemilloides*	Antioxidant and anti-inflammatory [70]	Wounds, sores, and abscesses [70]
*P. purpureum*	Antioxidant [71]	No specific ethnobotanical information available
*P. quercetorum*	Antioxidant and antibacterial [72]	Respiratory tract disorders
*P. inquinans*	Antioxidant [73]	Cold and bronchitis treatment [74]
*P. radula*	Antioxidant [75], antibacterial, and antifungal [76]	Diabetes treatment [75,76]
*P. hybrid*	Antioxidant, anti-inflammatory, anticholinesterase, and anti-haemolytic [77]	No specific ethnobotanical information available
*P. tabulare*	Antibacterial [78]	No specific ethnobotanical information available

**Table 2:** Selection of biological processes with higher score in the group G2 (*BP^G2^* > 0.85).

Biological Processes	*BP^mean^*	GO Frequency	*BP^G2^*
G protein-coupled acetylcholine receptor signaling pathway	0.81	0.01	0.89
Type II interferon-mediated signaling pathway	0.76	0.00	0.88
Regulation of cell adhesion mediated by integrin	0.77	0.01	0.88
Tachykinin receptor signaling pathway	0.82	0.01	0.87
Hormone catabolic process	0.79	0.00	0.87
Bradykinin catabolic process	0.79	0.00	0.87
Cellular response to virus	0.75	0.01	0.87
Positive regulation of the apoptotic signaling pathway	0.77	0.03	0.86
Positive regulation of the fatty acid metabolic process	0.76	0.01	0.86
Positive regulation of vasoconstriction	0.77	0.01	0.86
Response to pain	0.76	0.00	0.86
Cellular defense response	0.76	0.00	0.86
Response to dexamethasone	0.75	0.00	0.85
Smooth muscle contraction	0.76	0.02	0.85
Regulation of protein binding	0.77	0.00	0.85
Negative regulation of myosin-light-chain-phosphatase activity	0.75	0.00	0.85
Positive regulation of nitric oxide synthase biosynthetic process	0.73	0.00	0.85
Positive regulation of telomere capping	0.74	0.00	0.85
Regulation of inflammatory response	0.73	0.06	0.85

## Data Availability

The original contributions presented in this study are included in the article/Supplementary Material. Further inquiries can be directed to the corresponding authors.

## Supplementary Materials

The following supplementary materials can be downloaded at: https://www.mdpi.com/article/10.3390/molecules30153198/s1, Supplementary Materials 1. Comprehensive chemical and biological data for Pelargonium species. Supplementary Materials 2. Comprehensive analysis of biological activities in *Pelargonium* species.

## References

[1] Brendler T., van Wyk B.-E. (2008). A historical, scientific and commercial perspective on the medicinal use of *Pelargonium sidoides* (Geraniaceae). J. Ethnopharmacol..

[2] Manning J.C., Strlič M. (2024). *Pelargonium elizabethiae* and *P. geophyllum*, two new species in section *Hoarea* (Geraniaceae) from the Greater Cape Floristic Region, South Africa. S. Afr. J. Bot..

[3] van der Walt J.J.A. (1985). A taxonomic revision of the type section of *Pelargonium* L’Hérit. (Geraniaceae). Bothalia.

[4] Kolodziej H. (2011). Antimicrobial, antiviral and immunomodulatory activity studies of *Pelargonium sidoides* (EPs^®^ 7630) in the context of health promotion. Pharmaceuticals.

[5] van Wyk B.-E., Gericke N. (2000). People’s Plants: A Guide to Useful Plants of Southern Africa.

[6] Mativandlela S.P.N., Lall N., Meyer J.J.M. (2006). Antibacterial, antifungal and antitubercular activity of (the roots of) *Pelargonium reniforme* (CURT) and *Pelargonium sidoides* (DC) (Geraniaceae) root extracts. S. Afr. J. Bot..

[7] van Wyk B.-E. (2008). A review of Khoi-San and Cape Dutch medical ethnobotany. J. Ethnopharmacol..

[8] Sezik E., Yes E. (2001). Traditional medicine in Turkey X. Folk medicine in Central Anatolia. J. Ethnopharmacol..

[9] Scott G., Springfield E.P., Coldrey N. (2004). A Pharmacognostical study of 26 South African plant species used as traditional medicines. Pharm. Biol..

[10] Demarne F.E., van der Walt J.J.A. (1990). *Pelargonium tomentosum*: A potential source of peppermint-scented essential oil. S. Afr. J. Plant Soil.

[11] Ćavar S., Maksimović M. (2012). Antioxidant activity of essential oil and aqueous extract of *Pelargonium graveolens* L’Her. Food Control.

[12] Boukhatem M.N., Kameli A., Saidi F. (2013). Essential oil of Algerian rose-scented geranium (*Pelargonium graveolens*): Chemical composition and antimicrobial activity against food spoilage pathogens. Food Control.

[13] Hsouna A.B., Hamdi N. (2012). Phytochemical composition and antimicrobial activities of the essential oils and organic extracts from *Pelargonium graveolens* growing in Tunisia. Lipids Health Dis..

[14] Kolodziej H. (2007). Fascinating metabolic pools of *Pelargonium sidoides* and *Pelargonium reniforme*, traditional and phytomedicinal sources of the herbal medicine Umckaloabo^®^. Phytomedicine.

[15] Armijos C., Ramírez J., Vidari G. (2022). Poorly investigated ecuadorian medicinal plants. Plants.

[16] Pietta P., Simonetti P., Mauri P. (1998). Antioxidant Activity of Selected Medicinal Plants. J. Agric. Food Chem..

[17] Sen T., Samanta S.K. (2014). Medicinal plants, human health and biodiversity: A broad review. Adv. Biochem. Eng. Biotechnol..

[18] Van Wyngaard J., Famuyide I.M., Invernizzi L., Ndivhuwo K.K., Tordiffe A.S.W., Maharaj V.J., McGaw L.J. (2023). Optimizing extraction of *Pelargonium sidoides* roots: Impact of ethanol concentration on biological activity of extracts. S. Afr. J. Bot..

[19] Rezaizadehnajafi L., Wink M. (2014). EPs7630^®^ from *Pelargonium sidoides* increases stress resistance in *Caenorhabditis elegans* probably via the DAF-16/FOXO pathway. Phytomedicine.

[20] Lewu F.B., Grierson D.S., Afolayan A.J. (2006). Extracts from *Pelargonium sidoides* inhibit the growth of bacteria and fungi. Pharm. Biol..

[21] Samie S., Trollope K.M., Joubert L.M., Makunga N.P., Volschenk H. (2019). The antifungal and *Cryptococcus neoformans* virulence attenuating activity of *Pelargonium sidoides* extracts. J. Ethnopharmacol..

[22] Alossaimi M.A., Alzeer M.A., Abdel Bar F.M., ElNaggar M.H. (2022). *Pelargonium sidoides* root extract: Simultaneous HPLC separation, determination, and validation of selected biomolecules and evaluation of SARS-CoV-2 inhibitory activity. Pharmaceuticals.

[23] Lukman V., Odeyemi S.W., Roth R.L., Mbabala L., Tshililo N., Vlok N.M., Dewar M.J.B., Kenyon C.P. (2020). Novel kinase platform for the validation of the anti-tubercular activities of *Pelargonium sidoides* (Geraniaceae). BMC Biotechnol..

[24] Schnitzler P., Schneider S., Stintzing F.C., Carle R., Reichling J. (2008). Efficacy of an aqueous *Pelargonium sidoides* extract against herpesvirus. Phytomedicine.

[25] Kolodziej H., Kayser O., Radtke O.A., Kiderlen A.F., Koch E. (2003). Pharmacological profile of extracts of *Pelargonium sidoides* and their constituents. Phytomedicine.

[26] Bao Y., Gao Y., Koch E., Pan X., Jin Y., Cui X. (2015). Evaluation of pharmacodynamic activities of EPs^®^ 7630, a special extract from roots of *Pelargonium sidoides*, in animals models of cough, secretolytic activity and acute bronchitis. Phytomedicine.

[27] Janecki A., Conrad A., Engels I., Frank U., Kolodziej H. (2011). Evaluation of an aqueous-ethanolic extract from *Pelargonium sidoides* (EPs^®^ 7630) for its activity against group A-streptococci adhesion to human HEp-2 epithelial cells. J. Ethnopharmacol..

[28] Conrad A., Hansmann C., Engels I., Daschner F.D., Frank U. (2007). Extract of *Pelargonium sidoides* (EPs^®^ 7630) improves phagocytosis, oxidative burst, and intracellular killing of human peripheral blood phagocytes in vitro. Phytomedicine.

[29] Trun W., Kiderlen A.F., Kolodziej H. (2006). Nitric oxide synthase and cytokines gene expression analyses in *Leishmania*-infected RAW 264.7 cells treated with an extract of *Pelargonium sidoides* (Eps^®^ 7630). Phytomedicine.

[30] Thäle C., Kiderlen A., Kolodziej H. (2011). Anti-infective Activities of *Pelargonium sidoides* (EPS^®^ 7630): Effects of Induced NO Production on *Leishmania major* in Infected Macrophages and Antiviral Effects as Assessed in a Fibroblast-Virus Protection Assay. Planta Med..

[31] Mtimkulu Y., Lewu M.N., Mulidzi A.R., Lewu F. (2024). Cultivation and beneficial uses of *Pelargonium sidoides* DC.—A review. J. Med. Plants Econ. Dev..

[32] Zengin G., Leyva-Jiménez F.J., Fernández-Ochoa Á., Bouyahya A., Yildiztugay E., Carretero A.S., Mahomoodally M.F., Ponniya S.K.M., Nilofar, Koyuncu I. (2024). UHPLC-ESI-QTOF-MS metabolite profiles of different extracts from *Pelargonium endlicherianum* parts and their biological properties based on network pharmacological approaches. Arch. Pharm..

[33] Tepe B., Sokmen M., Akpulat H.A., Yumrutas O., Sokmen A. (2006). Screening of antioxidative properties of the methanolic extracts of *Pelargonium endlicherianum* Fenzl., *Verbascum wiedemannianum* Fisch. & Mey., *Sideritis libanotica* Labill. subsp. *linearis* (Bentham) Borm., *Centaurea mucronifera* DC. and *Hieracium cappadocicum* Freyn from Turkish flora. Food Chem..

[34] Ozbilge H., Kaya E.G., Taskin O.M., Kosar M. (2010). Antimicrobial activity of *Pelargonium endlicherianum* Fenzl. (Geraniaceae) roots against some microorganisms. J. Med. Plants Res..

[35] Cumaoğlu A., Karatoprak G.Ş., Yerer M.B., Koşar M. (2018). Anti-inflammatory effects of *Pelargonium endlicherianum* Fenzl. extracts in lipopolysaccharide-stimulated macrophages. Turk. J. Pharm. Sci..

[36] Kozan E., Küpeli Akkol E., Süntar I. (2016). Potential anthelmintic activity of *Pelargonium endlicherianum* Fenzl. J. Ethnopharmacol..

[37] Karatoprak G.Ş., Göger F., Yerer M.B., Koşar M. (2017). Chemical composition and biological investigation of *Pelargonium endlicherianum* root extracts. Pharm. Biol..

[38] Salem M.A., Radwan R.A., Mostafa E.S., Alseekh S., Fernie A.R., Ezzat S.M. (2020). Using an UPLC/MS-based untargeted metabolomics approach for assessing the antioxidant capacity and anti-aging potential of selected herbs. RSC Adv..

[39] Neagu A.F., Costea T., Nencu I., Duţu L.E., Popescu M.L., Olaru O.T., Gîrd C.E. (2018). Obtaining and characterization of a selective Pelargonium graveolens L’Hér. Dry extract with potential therapeutic activity in metabolic diseases. Farmacia.

[40] El Ouadi Y., Bendaif H., Mrabti H.N., Elmsellem H., Kadmi Y., Shariati M.A., Abdel-Rahman I., Hammouti B., Bouyanzer A. (2017). Antioxidant activity of phenols and flavonoids contents of aqueous extract of *Pelargonium graveolens* orgin in the North-East Morocco. J. Microbiol. Biotechnol. Food Sci..

[41] Al-Sayed E., Martiskainen O., Seif el-Din S.H., Sabra A.-N.A., Hammam O.A., El-Lakkany N.M. (2015). Protective effect of *Pelargonium graveolens* against carbon tetrachloride-induced hepatotoxicity in mice and characterization of its bioactive constituents by HPLC–PDA–ESI–MS/MS analysis. Med. Chem. Res..

[42] Saraswathi J., Prem K., Venkatesh K., Chakrapani P., Arun J.B., Amarashwari P., Sudhakar C., Roja Rani A. (2016). Evalution of qualitative analysis and antibacterial activity of *Pelargonium graveolens* L’Herit. Int. J. Phytomed..

[43] Pradeepa M., Kalidas V., Geetha N. (2016). Qualitative and quantitative phytochemical analysis and bactericidal activity of *Pelargonium graveolens* L’Her. Int. J. Appl. Pharm..

[44] Mahboubi M., Kazempour N., Farzin N. (2011). Antimicrobial activity of *Pelargonium graveolens* and *Oliveria decumbens* extracts against clinical isolates of *Staphylococcus aureus*. J. Biol. Act. Prod. Nat..

[45] Bayoub K., Baibai T., Mountassif D., Retmane A., Soukri A. (2010). Antibacterial activities of the crude ethanol extracts of medicinal plants against Listeria monocytogenes and some other pathogenic strains. Afr. J. Biotechnol..

[46] Amel H.A., Kamel H., Meriem F., Abdelkader K. (2022). Traditional Uses, Botany, Phytochemistry, and Pharmacology of *Pelargonium graveolens*: A Comprehensive Review. Trop. J. Nat. Prod. Res..

[47] Asgarpanah J., Ramezanloo F. (2015). An overview on phytopharmacology of *Pelargonium graveolens* L.. Indian J. Tradit. Knowl..

[48] Ben Hsouna A., Chahdoura H., Generalić Mekinić I., Maisto M., Kukula-Koch W., Ćavar Zeljković S., Koch W., Ben Akacha B., Taieb Bouteraa M., Ben Belgacem A. (2024). A comprehensive review on traditional uses, chemical composition, pharmacological effects and applications in the food industry of *Pelargonium odoratissimum* (L.) L’Hér. in comparison to other *Pelargonium* spp.. S. Afr. J. Bot..

[49] Andreou E., Triantafyllou A.K., Mountsaki S., Rallis E., Lamari F.N., Hatziantoniou S., Kefala V. (2022). Permanent make-up (PMU) inks decolorization using plant origin materials. Cosmetics.

[50] Iancu C., Cioancă O., Mircea C., Mocanu M., Hăncianu M. (2016). *Pelargonium* sp.: Characterization of the polyphenols and their biological potential. Farmacia.

[51] Iancu C., Filip N., Mocanu M., Dehelean C.A., Danciu C., Cioancă O., Hăncianu M., Corciovă A., Burlec F., Mircea C. (2023). Antioxidant and anticancer effect of some *Pelargonium* species extracts. Farmacia.

[52] Onduru Okeyo G., Charimbu M.K., Nyaanga J., Mendes T. (2022). Antibacterial activity of guava, moringa, camphor bush and pelargonium extracts against bacterial wilt (*Ralstonia pseudosolanacearum* sp. nov.) of potato. Saudi J. Biol. Sci..

[53] Iancu C., Cioancă O., Gaiddon C., Mircea C., Munteanu A., Filip N., Hanganu B., Manoilescu I., Hăncianu M. (2017). Cytoprotective and antiinflamatory activity evaluation of some *Pelargonium* extracts. Farmacia.

[54] Naz R., Saqib F., Latif M.F., Sajer B.H., Misarca C., Toma S.I., Podasca P.C., Andreescu O. (2025). Pharmacodynamic Elucidation of *Pelargonium zonale* (L.) L’Hér. ex Aiton for its Folkloric Claims in Diarrhea and Asthma via in Vitro, in Vivo and in Silico Methods. Nat. Prod. Commun..

[55] Páez X., Hernández L. (2003). Topical hemostatic effect of a common ornamental plant, the geraniaceae *Pelargonium zonale*. J. Clin. Pharmacol..

[56] Adewusi E.A., Afolayan A.J. (2010). Effect of *Pelargonium reniforme* roots on alcohol-induced liver damage and oxidative stress. Pharm. Biol..

[57] Adewusi E.A., Afolayan A.J. (2009). Antibacterial, antifungal and antioxidant activity of the roots and leaves of *Pelargonium reniforme* Curtis (Geraniaceae). Afr. J. Biotechnol..

[58] Kim C.E., Griffiths W.J., Taylor P.W. (2009). Components derived from *Pelargonium* stimulate macrophage killing of *Mycobacterium* species. J. Appl. Microbiol..

[59] Kolodziej H., Kiderlen A.F. (2007). In vitro evaluation of antibacterial and immunomodulatory activities of *Pelargonium reniforme*, *Pelargonium sidoides* and the related herbal drug preparation EPs^®^ 7630. Phytomedicine.

[60] Iancu C., Mircea C., Pietrariu F., Cioancă O., Stan C., Corciovă A., Murărașu A., Filip N., Hăncianu M. (2020). The evaluation of normo-glycemic and cyto-regenerative effects of *Pelargonium* species extracts. Farmacia.

[61] Coronado-López S., Caballero-García S., Aguilar-Luis M.A., Mazulis F., del Valle-Mendoza J. (2018). Antibacterial activity and cytotoxic effect of *Pelargonium peltatum* (Geranium) against *Streptococcus mutans* and *Streptococcus sanguinis*. Int. J. Dent..

[62] Guerrero J., Ortiz Z., Peralta L., Pérez F. (2013). Antibacterial activity of *Pelargonium peltatum* (L.) L’Her. against *Streptococcus mutans*, *Streptococcus sanguis* and *Streptococcus mitis* versus chlorhexidine. Rev. Cuba. Plantas Med..

[63] Rungqu P., Oyedeji O., Gondwe M., Oyedeji A. (2023). Chemical Composition, Analgesic and Anti-Inflammatory Activity of *Pelargonium peltatum* Essential Oils from Eastern Cape, South Africa. Molecules.

[64] Maroufi Y., Hoseini S.R., Alavi M. (2022). Antiparasitic effect of leaf extract and major metabolites of *Pelargonium quercetorum* Agnew. against *Leishmania Major*: In Vitro and In Silico studies. J. Appl. Biotechnol. Rep..

[65] Aztopal N., Cevatemre B., Sarimahmut M., Ari F., Dere E., Ozel M.Z., Firat M., Ulukaya E. (2016). *Pelargonium quercetorum* Agnew induces apoptosis without PARP or cytokeratin 18 cleavage in non-small cell lung cancer cell lines. Oncol. Lett..

[66] Cock I.E., Orchard A., Booi L., van Vuuren S.F. (2024). Southern African traditional herbal medicinal plants used to treat cardiovascular disease and related medical conditions: Traditional use and scientific evidence. S. Afr. J. Bot..

[67] Pranskuniene Z., Dauliute R., Pranskunas A., Bernatoniene J. (2018). Ethnopharmaceutical knowledge in Samogitia region of Lithuania: Where old traditions overlap with modern medicine. J. Ethnobiol. Ethnomed..

[68] Kaval I., Behçet L., Cakilcioglu U. (2014). Ethnobotanical study on medicinal plants in Geçitli and its surrounding (Hakkari-Turkey). J. Ethnopharmacol..

[69] Kujawska M., Klepacki P., Łuczaj Ł. (2017). Fischer’s Plants in folk beliefs and customs: A previously unknown contribution to the ethnobotany of the Polish-Lithuanian-Belarusian borderland. J. Ethnobiol. Ethnomed..

[70] Mbhele N., Ncube B., Ndhlala A.R., Moteetee A. (2022). Pro-inflammatory enzyme inhibition and antioxidant activity of six scientifically unexplored indigenous plants traditionally used in South Africa to treat wounds. S. Afr. J. Bot..

[71] Koutelidakis A.E., Serafini M., Komaitis M., Kapsokefalou M. (2010). Oxidative activity of some iron compounds on colon tissue homogenates from mice after administration of green tea, white tea and *Pelargonium purpureum*. Food Chem..

[72] Fernandez-Soto P., Celi D., Tejera E., Alvarez-Suarez J.M., Machado A. (2023). *Cinnamomum* sp. and *Pelargonium odoratissimum* as the main contributors to the antibacterial activity of the medicinal drink horchata: A study based on the antibacterial and chemical analysis of 21 plants. Molecules.

[73] Izuegbuna O., Otunola G., Bradley G. (2019). Estimation of phytochemical, vitamins composition and antioxidant activity of *Pelargonium inquinans* leaves. Pharmacogn. J..

[74] Tembeni B., Oyedeji O.O., Manene C.N., Oyemitan I.A., Oyedeji A.O. (2019). Anti-inflammatory, Analgesic Activity and Toxicity of Two *Pelargonium inquinans* Ait Essential Oils: Wild and Cultivated. J. Essent. Oil-Bear. Plants.

[75] Petlevski R., Flajs D., Kalodera Z., Zovko Končić M. (2013). Composition and antioxidant activity of aqueous and ethanolic *Pelargonium radula* extracts. S. Afr. J. Bot..

[76] Pepeljnjak S., Kalodera Z., Zovko M. (2005). Investigation of antimicrobial activity of *Pelargonium radula* (Cav.) L’Hérit. Acta Pharm..

[77] Fayoumi L., Khalil M., Ghareeb D., El-Dakdouki M.H. (2022). Chemical composition and therapeutic activity of lebanese rose geranium (*Pelargonium* hybrid) extracts. Farmacia.

[78] Jantová S., Nagy M., Ružeková Ĺ., Grančai D. (2000). Antibacterial activity of plant extracts from the families Fabaceae, Oleaceae, Philadelphaceae, Rosaceae and Staphyleaceae. Phytother. Res..

[79] Beltrán-Noboa A., Jordan-Álvarez A., Guevara-Terán M., Gallo B., Berrueta L.A., Giampieri F., Battino M., Álvarez-Suarez J.M., Tejera E. (2023). Exploring the chemistry of *Ocimum* species under specific extractions and chromatographic methods: A systematic review. ACS Omega.

[80] Abdelbaky A.S., Abd El-Mageed T.A., Babalghith A.O., Selim S., Mohamed A.M.H.A. (2022). Green synthesis and characterization of ZnO nanoparticles using *Pelargonium odoratissimum* (L.) aqueous leaf extract and their antioxidant, antibacterial and anti-inflammatory activities. Antioxidants.

[81] Alqahtani A.A., El Raey M.A., Abdelsalam E., Ibrahim A.M., Alqahtani O., Torky Z.A., Attia H.G. (2022). The biosynthesized zinc oxide nanoparticles’ antiviral activity in combination with *Pelargonium zonale* extract against the human Corona 229E virus. Molecules.

[82] Venter A., Fisher H., Stafford G.I., Duodu K.G. (2022). Pigmented flower extracts of plant species from the Geraniaceae and Lamiaceae families as natural food colourants: Anthocyanin composition, thermal and oxidative stability. Int. J. Food Sci. Technol..

[83] Williams C.A., Newman M., Gibby M. (2000). The application of leaf phenolic evidence for systematic studies within the genus *Pelargonium* (Geraniaceae). Biochem. Syst. Ecol..

[84] Judge M.T., Ebbels T.M.D. (2022). Problems, principles and progress in computational annotation of NMR metabolomics data. Metabolomics.

[85] Chaleckis R., Meister I., Zhang P., Wheelock C.E. (2019). Challenges, progress and promises of metabolite annotation for LC–MS-based metabolomics. Curr. Opin. Biotechnol..

[86] Weinstein M.P., Lewis II J.S., Bobenchik A.M., Campeau S., Cullen S.K., Galas M.F., Gold H., Humphries R.M., Kirn T.J., Limbago B. (2020). Performance Standards for Antimicrobial Susceptibility Testing.

[87] Matuschek E., Brown D.F.J., Kahlmeter G. (2014). Development of the EUCAST disk diffusion antimicrobial susceptibility testing method and its implementation in routine microbiology laboratories. Clin. Microbiol. Infect..

[88] Aoac International (2012). AOAC SMPR 2011.011 Standard Method Performance Requirements for in vitro Determination of Total Antioxidant Activity in Foods, Beverages, Food Ingredients, and Dietary Supplements. J. AOAC Int..

[89] Scott K.A., Ropek N., Melillo B., Schreiber S.L., Cravatt B.F., Vinogradova E.V. (2022). Stereochemical diversity as a source of discovery in chemical biology. Curr. Res. Chem. Biol..

[90] Careddu D., Pettenazzo A. (2018). *Pelargonium sidoides* extract EPs 7630: A review of its clinical efficacy and safety for treating acute respiratory tract infections in children. Int. J. Gen. Med..

[91] Moyo M., Van Staden J. (2014). Medicinal properties and conservation of *Pelargonium sidoides* DC. J. Ethnopharmacol..

[92] Wopker P.M., Schwermer M., Sommer S., Längler A., Fetz K., Ostermann T., Zuzak T.J. (2020). Complementary and alternative medicine in the treatment of acute bronchitis in children: A systematic review. Complement. Ther. Med..

[93] Nöldner M., Schötz K. (2007). Inhibition of lipopolysaccharide-induced sickness behavior by a dry extract from the roots of *Pelargonium sidoides* (EPs^®^ 7630) in mice. Phytomedicine.

[94] Duarte J., Francisco V., Perez-Vizcaino F. (2014). Modulation of nitric oxide by flavonoids. Food Funct..

[95] Lee J.H., Kim G.H. (2010). Evaluation of antioxidant and inhibitory activities for different subclasses flavonoids on enzymes for rheumatoid arthritis. J. Food Sci..

[96] Sadik C.D., Sies H., Schewe T. (2003). Inhibition of 15-lipoxygenases by flavonoids: Structure-activity relations and mode of action. Biochem. Pharmacol..

[97] Chen Y., Shen S., Lee W., Hou W., Yang L., Lee T.J.F. (2001). Inhibition of nitric oxide synthase inhibitors and lipopolysaccharide induced inducible NOS and cyclooxygenase-2 gene expressions by rutin, quercetin, and quercetin pentaacetate in RAW 264.7 macrophages. J. Cell Biochem..

[98] Maldonado-Rojas W., Olivero-Verbel J. (2012). Food-related compounds that modulate expression of inducible nitric oxide synthase may act as its inhibitors. Molecules.

[99] Larit F., León F., Benyahia S., Cutler S.J. (2019). Total phenolic and flavonoid content and biological activities of extracts and isolated compounds of *Cytisus villosus* pourr. Biomolecules.

[100] Giridharan S., Srinivasan M. (2018). Mechanisms of NF-κB p65 and strategies for therapeutic manipulation. J. Inflamm. Res..

[101] Choy K.W., Murugan D., Leong X.-F., Abas R., Alias A., Mustafa M.R. (2019). Flavonoids as natural anti-inflammatory agents targeting nuclear Factor-Kappa B (NFκB) signaling in cardiovascular diseases: A Mini Review. Front. Pharmacol..

[102] Ramanan M., Sinha S., Sudarshan K., Aidhen I.S., Doble M. (2016). Inhibition of the enzymes in the leukotriene and prostaglandin pathways in inflammation by 3-aryl isocoumarins. Eur. J. Med. Chem..

[103] Sudarshan K., Aidhen I.S. (2017). Convenient Synthesis of 3-Glycosylated Isocoumarins. Eur. J. Org. Chem..

[104] Khodzhaieva R.S., Gladkov E.S., Kyrychenko A., Roshal A.D. (2021). Progress and Achievements in Glycosylation of Flavonoids. Front. Chem..

[105] Tang S., Wang B., Liu X., Xi W., Yue Y., Tan X., Bai J., Huang L. (2025). Structural insights and biological activities of flavonoids: Implications for novel applications. Food Front..

[106] Flores-Morales V., Villasana-Ruíz A.P., Garza-Veloz I., González-Delgado S., Martinez-Fierro M.L. (2023). Therapeutic Effects of Coumarins with Different Substitution Patterns. Molecules.

[107] Hollman P.C.H. (2004). Absorption, Bioavailability, and Metabolism of Flavonoids. Pharm. Biol..

[108] Hollman P.C.H., Bijsman M.N.C.P., van Gameren Y., Cnossen E.P.J., de Vries J.H.M., Katan M.B. (1999). The sugar moiety is a major determinant of the absorption of dietary flavonoid glycosides in man. Free Radic. Res..

[109] Chen L., Cao H., Huang Q., Xiao J., Teng H. (2022). Absorption, metabolism and bioavailability of flavonoids: A review. Crit. Rev. Food Sci. Nutr..

[110] Thilakarathna S.H., Vasantha Rupasinghe H.P. (2013). Flavonoid bioavailability and attempts for bioavailability enhancement. Nutrients.

[111] Chen L., Lin X., Fan X., Qian Y., Lv Q., Teng H. (2020). Sonchus oleraceus Linn extract enhanced glucose homeostasis through the AMPK/Akt/ GSK-3β signaling pathway in diabetic liver and HepG2 cell culture. Food Chem. Toxicol..

[112] Silva P., Bonifácio B., Ramos M., Negri K., Maria Bauab T., Chorilli M. (2013). Nanotechnology-based drug delivery systems and herbal medicines: A review. Int. J. Nanomed..

[113] Caddeo C., Gabriele M., Fernàndez-Busquets X., Valenti D., Fadda A.M., Pucci L., Manconi M. (2019). Antioxidant activity of quercetin in Eudragit-coated liposomes for intestinal delivery. Int. J. Pharm..

[114] Ciaraldi T.P., Nikoulina S.E., Bandukwala R.A., Carter L., Henry R.R. (2007). Role of glycogen synthase kinase-3α in insulin action in cultured human skeletal muscle cells. Endocrinology.

[115] Wang L., Li J., Di L. (2022). Glycogen synthesis and beyond, a comprehensive review of GSK3 as a key regulator of metabolic pathways and a therapeutic target for treating metabolic diseases. Med. Res. Rev..

[116] Sun A., Simsek Papur O., Dirkx E., Wong L., Sips T., Wang S., Strzelecka A., Nabben M., Glatz J.F.C., Neumann D. (2021). Phosphatidylinositol 4-kinase IIIβ mediates contraction-induced GLUT4 translocation and shows its anti-diabetic action in cardiomyocytes. Cell. Mol. Life Sci..

[117] Liu L., Zhang Y., Wang L., Liu Y., Chen H., Hu Q., Xie C., Meng X., Shen X. (2023). Scutellarein alleviates chronic obstructive pulmonary disease through inhibition of ferroptosis by chelating iron and interacting with arachidonate 15-lipoxygenase. Phytother. Res..

[118] Hosseinymehr M., Matin M.M., Sadeghian H., Bahrami A.R., Kaseb-Mojaver N. (2016). 8-Farnesyloxycoumarin induces apoptosis in PC-3 prostate cancer cells by inhibition of 15-lipoxygenase-1 enzymatic activity. Anticancer Drugs.

[119] Sun Q., Lv M., Wang Z. (2025). Delphinidin inhibits the ALOX15-mediated ferroptosis in rats to alleviate myocardial ischemia and reperfusion injury. Biochim. Biophys. Acta (BBA)—Mol. Cell Res..

[120] Arita M., Philipov S., Galabov A.S. (2015). Phosphatidylinositol 4-kinase III beta is the target of oxoglaucine and pachypodol (Ro 09-0179) for their anti-poliovirus activities, and is located at upstream of the target step of brefeldin A. Microbiol. Immunol..

[121] Kim H.W., Wang M., Leber C.A., Nothias L.-F., Reher R., Kang K.B., van der Hooft J.J.J., Dorrestein P.C., Gerwick W.H., Cottrell G.W. (2021). NPClassifier: A deep neural network-based structural classification tool for natural products. J. Nat. Prod..

[122] Beltrán-Noboa A., Proaño-Ojeda J., Guevara M., Gallo B., Berrueta L.A., Giampieri F., Perez-Castillo Y., Battino M., Álvarez-Suarez J.M., Tejera E. (2022). Metabolomic profile and computational analysis for the identification of the potential anti-inflammatory mechanisms of action of the traditional medicinal plants, *Ocimum basilicum* and *Ocimum tenuiflorum*. Food Chem. Toxicol..

[123] Jimenes-Vargas K., Pazos A., Munteanu C.R., Perez-Castillo Y., Tejera E. (2024). Prediction of compound-target interaction using several artificial intelligence algorithms and comparison with a consensus-based strategy. J. Cheminform.

[124] Guo Y., Bruno R.S. (2015). Endogenous and exogenous mediators of quercetin bioavailability. J. Nutr. Biochem..

[125] Petersen B., Egert S., Bosy-Westphal A., Müller M.J., Wolffram S., Hubbermann E.M., Rimbach G., Schwarz K. (2016). Bioavailability of quercetin in humans and the influence of food matrix comparing quercetin capsules and different apple sources. Food Res. Int..

[126] Huang D.W., Sherman B.T., Lempicki R.A. (2009). Systematic and integrative analysis of large gene lists using DAVID bioinformatics resources. Nat. Protoc..

[127] Supek F., Bošnjak M., Škunca N., Šmuc T. (2011). Revigo summarizes and visualizes long lists of gene ontology terms. PLoS ONE.

