# Supplementary material for: Chemical Composition and Biological Activities of Pelargonium sp.: A Review with In Silico Insights into Potential Anti-Inflammatory Mechanism
Source: Molecules. 2025 Jul 30;30(15):3198. doi: 10.3390/molecules30153198 (PMC12348979; doi:10.3390/molecules30153198)
Supplement: Supplementary file 1 [file molecules-30-03198-s001.zip › molecules-3746657-Supplementary Materials 2.pdf]

Supplementary Materials

Chemical Composition and Biological Activities of *Pelargonium* sp.: A Review with In Silico Insights into Potential Anti-Inflammatory Mechanism

Diana Celi [1], Karina Jimenes-Vargas [2,3], António Machado [4,5], José Miguel Álvarez-Suárez [6,7,*] and Eduardo Tejera [1,2,*]

[1] Facultad de Ingeniería y Ciencias Aplicadas, Universidad de Las Américas (UDLA), Quito 170504, Ecuador
[2] Bio-Cheminformatics Research Group, Universidad de Las Américas (UDLA), Quito 170504, Ecuador
[3] Departament of Computer Science and Information Technologies, Faculty of Computer Science, Universidade da Coruña, Campus Elviña s/n, 15071 A Coruña, Spain
[4] Centro de Biotecnologia dos Açores (CBA), Departamento de Biologia, Faculdade de Ciências e Tecnologia, Universidade dos Açores, 9500-321 Ponta Delgada, Portugal
[5] Laboratorio de Bacteriología, Instituto de Microbiología, Colegio de Ciencias Biológicas y Ambientales COCIBA, Universidad San Francisco de Quito (USFQ), Quito 170901, Ecuador
[6] Laboratorio de Investigación en Ingeniería en Alimentos (LabInAli), Departamento de Ingeniería en Alimentos, Colegio de Ciencias e Ingenierías, Universidad San Francisco de Quito (USFQ), Quito 170901, Ecuador
[7] Laboratorio de Bioexploración, Colegio de Ciencias Biológicas y Ambientales, Universidad San Francisco de Quito (USFQ), Quito 170901, Ecuador
* Correspondence: jalvarez@usfq.edu.ec (J.M.Á.-S.); eduardo.tejera@udla.edu.ec (E.T.)

Academic Editors: Kuniyoshi Shimizu and Yhiya Amen

Received: 24 June 2025
Revised: 18 July 2025
Accepted: 23 July 2025
Published: 30 July 2025

Citation: Celi, D.; Jimenes-Vargas, K.; Machado, A.; Álvarez-Suárez, J.M.; Tejera, E. Chemical Composition and Biological Activities of *Pelargonium* sp.: A Review with In Silico Insights into Potential Anti-Inflammatory Mechanism. *Molecules* **2025**, *30*, 3198. https://doi.org/10.3390/molecules30153198

Copyright: © 2025 by the authors. Licensee MDPI, Basel, Switzerland. This article is an open access article distributed under the terms and conditions of the Creative Commons Attribution (CC BY) license (https://creativecommons.org/licenses/by/4.0/).

1. Biological activities

1.1. Antioxidant activity

Antioxidant activity is defined as the capacity of specific molecules or chemical compounds to neutralize or mitigate the detrimental effects of free radicals and other reactive oxygen species within the body. A number of studies have demonstrated that extracts derived from various *Pelargonium* species exhibit remarkable antioxidant properties. Moreover, a variety of antioxidant capacity assays have been utilized by researchers to evaluate the potency of these extracts.

For example, *P. zonale* has demonstrated notable efficacy in neutralizing superoxide radicals (IC_{50} = 18.34 µg/mL) and hydroxyl radicals (IC_{50} = 12.46 µg/mL), exhibiting a superior performance compared to *P. radens*, which exhibited IC_{50} values of 95.83 µg/mL and 24.563 µg/mL, respectively. In iron chelation studies, *P. zonale* demonstrated an IC_{50} of 77.06 µg/mL, while *P. radens* exhibited superior performance with an IC_{50} of 30.46 µg/mL. In comparison to the standard gallic acid, *P. zonale* exhibited superior antioxidant activity in superoxide and hydroxyl radical assays. However, it demonstrated slightly diminished efficacy in iron chelation, where gallic acid exhibited an IC_{50} value of 19.14 µg/mL [51]. Moreover, in DPPH, ABTS, and reducing power assays, *P. zonale* exhibited IC_{50} values of 4.69 mg/mL, 99.51 mg/mL, and 124.64 mg/mL, respectively. In the same assays, the antioxidant activity of *P. zonale* demonstrated significantly superior performance, with IC_{50} values of 4.69 mg/mL, 99.51 mg/mL, and 124.64 mg/mL, respectively, in the same assays, which were significantly higher than those observed for *P. hispidum* (14.11 mg/mL, 176.99 mg/mL, and 448.63 mg/mL). The enhanced antioxidant activity of *P. zonale* can be attributed to its elevated polyphenol and flavonoid content, including catechin, quercetin, and cyanidin, which are renowned for their potent free radical scavenging capabilities.

Salem et al. [38] evaluated the antioxidant capacity of 95% ethanolic and aqueous extracts of *P.* graveolens using DPPH and ORAC assays, with ascorbic acid and Trolox as

standards. The IC_{50} values were approximately 11 μg/mL for the ethanolic extract and 20 μg/mL for the aqueous extract in the DPPH assay. These values, though not optimal, are comparable to those observed for *Camellia sinensis* and *Matricaria chamomilla*. In the ORAC assay, *P. graveolens* (IC_{50} of 11 and 18 μg/mL for aqueous and ethanolic extracts, respectively) exhibited superior antioxidant activity compared to Trolox (IC_{50} of approximately 28 μg/mL). These findings indicate that *P. graveolens*, while not exhibiting the highest antioxidant capacity within the study, displays a notable ability to neutralize free radicals. These findings are further supported by the work of Neagu et al. [39] , who evaluated the antioxidant capacity of a dry extract of *P. graveolens* using the ABTS and ferric reducing antioxidant power (FRAP) methods. The IC_{50} was determined to be 17.53 μg/mL for ABTS and 74.43 μg/mL for the reducing iron power. The extract exhibited a superior antioxidant capacity relative to ascorbic acid in the ABTS assay, indicating a notable antioxidant potential associated with its high phenol and anthocyanin content.

Moreover, *P. endlicherianum* has been subjected to a comprehensive evaluation by Zengin et al. [32] using six different methods: The antioxidant capacity of the plant was evaluated using several methods, including DPPH, ABTS, FRAP, CUPRAC, phosphomolybdenum, and metal chelation. A variety of extracts, including those derived from ethyl acetate, dichloromethane, ethanol, and water, were obtained from both the aerial parts and roots of the plant. The findings revealed that aqueous and ethanolic extracts of the aerial parts exhibited remarkable antioxidant capacity, particularly in the DPPH (774.35 mg TE/g for water and 713.03 mg TE/g for ethanol) and ABTS (1257.28 mg TE/g for water) assays. Additionally, the root's ethanolic fractions exhibited notable antioxidant activity in the ABTS method, with a TE/g value of 1350. In the FRAP and CUPRAC assays, the aqueous and ethanolic extracts of the aerial parts exhibited high activity, with the ethanolic extract of water demonstrating a notable 842.47 TE/g in the FRAP assay. The phosphomolybdenum method indicated high activity in the aqueous and ethanolic extracts of the aerial parts, while the ethyl acetate extracts from both parts demonstrated notable metal chelation efficacy. The study identified a correlation between elevated antioxidant capacity and higher phenolic content, indicating that *P. endlicherianum* may be a promising source of natural antioxidants.

Rezaizadehnajafi and Wink [19] examined the EPS® 7630 extract, utilizing the DPPH assay to ascertain the extract's capacity to scavenge free radicals. The results demonstrated that EPS® 7630 exhibited notable antioxidant properties, with an IC_{50} value of 14.7 μg/mL. This was compared to the epigallocatechin-3-gallate control, which demonstrated an IC_{50} of 4.24 μg/mL. Although the extract demonstrated a less pronounced antioxidant activity than the control, it exhibited notable free radical scavenging capabilities.

In a comparative study conducted by Mbhele et al. [70], the antioxidant capacity of *P. alchemilloides* was evaluated alongside that of five additional medicinal plants native to South Africa. The following plants were included in the study: *Agapanthus inapertus*, *Cheilanthes hirta*, *Crassula capitella*, *Eriospermum flagelliforme*, and *Euphorbia clavarioides.* The antioxidant capacity of the extracts was evaluated through the application of four distinct methodologies: The antioxidant capacity of the extracts was evaluated by four different methods: DPPH, NO, FRAP, and β-carotene-linoleic acid (β-CLAMS). *P. alchemilloides* exhibited a notable performance in the DPPH assay, with its aqueous extract displaying a noteworthy radical scavenging capacity (IC_{50} of 0.07 mg/mL), surpassing that of ascorbic acid (IC_{50} = 0.23 mg/mL) and approaching that of butylated hydroxytoluene (BHT, IC_{50} = 0.05 mg/mL), which were the standards utilized in this investigation. Nevertheless, in all the employed methods, *C. capitella* exhibited superior performance.

Moreover, Izuegbuna et al. [73] conducted studies employing the DPPH, nitric oxide, hydrogen peroxide, and phosphomolybdenum assays for *P. inquinans*. The research findings indicated that both aqueous and ethanolic extracts exhibited notable antioxidant

capacity. For nitric oxide scavenging activity, the aqueous extract showed an IC_{50} of 0.741 mg/mL and the ethanol extract 0.805 mg/mL, both outperforming vitamin C and gallic acid (1.18 mg/mL). In DPPH scavenging, the aqueous extract had an IC_{50} of 0.461 mg/mL, and the ethanol extract 0.476 mg/mL, closely following the standards (0.436 mg/mL for vitamin C and 0.439 mg/mL for gallic acid). The phosphomolybdenum antioxidant assay revealed an IC_{50} of 0.293 mg/mL for the aqueous extract, which was comparable to vitamin C (0.296 mg/mL) and gallic acid (0.298 mg/mL), while the ethanol extract had a higher IC_{50} of 0.344 mg/mL. For hydrogen peroxide scavenging, the aqueous extract had an IC_{50} of 0.381 mg/mL, and the ethanol extract 0.397 mg/mL, slightly less effective than vitamin C (0.376 mg/mL) and gallic acid (0.378 mg/mL). These findings lend support to the traditional medicinal use of *P. inquinans* in the treatment of various ailments and suggest its potential as a natural source of antioxidants.

Lastly, in our research group [72], we demonstrated that the *P. odoratissimum* extract exhibited remarkable antioxidant capacity in both the DPPH and FRAP assays, when compared with the 21 plants that are used in the horchata drink. In the DPPH and FRAP assays, the extract demonstrated antioxidant activity of 2777.9 μmol TEq/g DE and 901.8 μmol TEq/g DE, respectively. We observed a strong correlation between this notable antioxidant capacity and the total phenol content (TPC) of the extract, indicating that phenolic compounds are a primary contributor to the antioxidant activity of *P. odoratissimum.* **Table 1** presents a synopsis of supplementary research on the antioxidant properties of several plant crude extracts derived from *Pelargonium* species.

Table 1. Antioxidant activities of different crude extracts from *Pelargonium* plant species.

Pelargonium Source	Plant extract	Part plant	Mechanism	Doses/ Concentration/ Inhibition %	Reference
P. graveolens	Ethanolic 95%	Aerial parts	ORAC	IC_{50} = 18 (mg/mL)	[38]
			DPPH	IC_{50} = 11 (mg/mL)	
	Aqueous		ORAC	IC_{50} = 12 (mg/mL)	
			DPPH	IC_{50} = 20 (mg/mL)	
	Ethanolic 50%	Leaves, flowers, and aerial parts	ABTS	IC_{50} = 17.53 (mg/mL)	[39]
			FRP	IC_{50} = 4.43 (mg/mL)	
P. zonale	Methanolic	Leaves	FIC	IC_{50} = 77.06 (mg/mL)	[51]
			O_2	IC_{50} = 18.34 (mg/mL)	
			OH	IC_{50} = 12.46 (mg/mL)	
	Aqueous	Leaves	DPPH	20.87 (μg TE/mL)	[49]
			FRAP	3.56 (mg $FeSO_4 \times 7H_2O$/mL)	
	Methanolic	Leaves	DPPH	IC_{50} = 4.69 (mg/mL)	[50]
			ABTS	IC_{50} = 99.51 (mg/mL)	
			FRP	IC_{50} = 124.64 (mg/mL)	
P. sidoides	EPS® 7630	Roots	ABTS	IC_{50} = 37.14 (mg/mL)	[18]
	Ethanolic 60 %			IC_{50} = 97.70 (mg/mL)	
	EPS® 7630		DPPH	IC_{50} = 4.34 (mg/mL)	[19]
P. endlicherianum	Aqueous	Aerial parts	DPPH	774.35 (mg TE/g)	[32]
			ABTS	1257.28 (mg TE/g)	

			CUPRAC	669.18 (mg TE/g)	
			FRAP	842.47 (mg TE/g)	
			PBD	2.58 (mmol TE/g)	
			MCA	26.20 (mg EDTAE/g)	
		Roots	DPPH	224.44 (mg TE/g)	
			ABTS	499.38 (mg TE/g)	
			CUPRAC	243.03 (mg TE/g)	
			FRAP	273.99 (mg TE/g)	
			PBD	1.72 (mmol TE/g)	
			MCA	29.89 (mg EDTAE/g)	
	Ethanolic	Aerial parts	DPPH	713.03 (mg TE/g)	
			ABTS	1170.78 (mg TE/g)	
			CUPRAC	645.88 (mg TE/g)	
			FRAP	671.09 (mg TE/g)	
			PBD	2.81 (mmol TE/g)	
			MCA	21.33 (mg EDTAE/g)	
		Roots	DPPH	441.80 (mg TE/g)	
			ABTS	1350.00 (mg TE/g)	
			CUPRAC	510.41 (mg TE/g)	
			FRAP	551.78 (mg TE/g)	
			PBD	2.72 (mmol TE/g)	
			MCA	5.14 (mg EDTAE/g)	
	Methanolic	Whole plant	DPPH	IC_{50} = 7.43 (mg/mL)	[33]
			β-Carotene-linoleic acid	72.6 %	
P. alchemilloides	Aqueous	Whole plant	DPPH	IC_{50} = 0.07 (mg/mL)	[70]
			NO	IC_{50} = 0.75 (mg/mL)	
	Ethanolic 70%		DPPH	IC_{50} = 0.16 (mg/mL)	
			NO	IC_{50} = 0.61 (mg/mL)	
	Methanolic 50%		DPPH	IC_{50} = 0.19 (mg/mL)	
			NO	IC_{50} = 9.72 (mg/mL)	
P. inquinans	Aqueous	Leaves	DPPH	IC_{50} = 0.461 (mg/mL)	[73]
			NO	IC_{50} = 0.741 (mg/mL)	
			H_2O_2	IC_{50} = 0.381 (mg/mL)	
			PBD	IC_{50} = 0.293 (mg/mL)	
	Ethanolic		DPPH	IC_{50} = 0.476 (mg/mL)	
			NO	IC_{50} = 0.805 (mg/mL)	
			H_2O_2	IC_{50} = 0.476 (mg/mL)	
			PBD	IC_{50} = 0.397 (mg/mL)	
P. hybrid	Aqueous	Leaves	DPPH	IC_{50} = 10.1 (μg/mL)	[77]
			FRP	IC_{50} = 316.4 (μg/mL)	
			β-Carotene bleaching	IC_{50} = 103 (μg/mL)	

		Stems	DPPH	IC_{50} = 28.3 (μg/mL)	
			FRP	IC_{50} = 795.5 (μg/mL)	
			β-Carotene bleaching	IC_{50} = 410.2 (μg/mL)	
	Ethanolic	Leaves	DPPH	IC_{50} = 7.88 (μg/mL)	
			H_2O_2	IC_{50} = 2533 (μg/mL)	
			FRP	IC_{50} = 143.3 (μg/mL)	
			β-Carotene bleaching	IC_{50} = 78.3 (μg/mL)	
		Stems	DPPH	IC_{50} = 10.0 (μg/mL)	
			H_2O_2	IC_{50} = 3550 (μg/mL)	
			FRP	IC_{50} = 137.2 (μg/mL)	
			β-Carotene bleaching	IC_{50} = 533.4 (μg/mL)	
P. hispidum	Methanolic	Leaves	DPPH	IC_{50} = 14.11 (μg/mL)	[50]
			ABTS	IC_{50} = 176.99 (μg/mL)	
			FRP	IC_{50} = 448.63 (μg/mL)	
P. odaratissimum	Methanolic 80%	Plant without root	DPPH	901.7 (μmol TE/g)	[72]
			FRAP	2777.9 (μmol TE/g)	
P. radens	Methanolic	Leaves	FIC	IC_{50} = 30.46 (μg/mL)	[72]
			O_2	IC_{50} = 95.83 (μg/mL)	
			OH	IC_{50} = 17.94 (μg/mL)	

ORAC: Oxygen radical absorbance capacity, DPPH: 2,2-diphenyl-1-picrylhydrazyl, ABTS: (2,2′ - azinobis-(3-ethylbenzothiazoline-6-sulfonic acid, FRAP: Ferric reducing power, FIC: Ferrous ion–chelating, CUPRAC: Cupric reducing antioxidant capacity, PBD: Phosphomolybdenum assay, MCA: Metal chelating assay, NO: nitric oxide, O_2: Superoxide anion radical scavenging, OH: Hydroxyl radical, TE: Trolox equivalent.

One problem we encountered as a group when analyzing and comparing antioxidant capacity is the inconsistency in the units of measurement used in different studies. In some cases, antioxidant activity is measured as IC_{50}, in others as a percentage of inhibition and in others as equivalents of the standard per gram or milliliter of the extract (even changing the type of standard). This variability in measurement units makes it difficult to directly compare results and interpret the antioxidant efficacy of the analyzed extracts. In other words, it is difficult to carry out a meta-analysis for species comparison.

1.2. Antibacterial Activity

The antibacterial activity of a molecule is closely related to its capacity to destroy or hinder the growth of bacteria while minimizing damage to nearby tissues. Numerous contemporary antimicrobial agents originate from natural compounds that have been chemically altered. Numerous studies have shown that extracts from different *Pelargonium* species possess notable antibacterial properties. In our analysis, we concentrated exclusively on studies that provided minimum inhibitory concentration (MIC) values to evaluate the efficacy of these extracts against a range of bacterial pathogens.

Kolodziej et al. [25] investigated the antibacterial efficacy of EPS® 7630 against multiresistant strains of *Staphylococcus aureus*. Although the effect was moderate compared to *S. aureus* ATCC 25923, the extract showed remarkable antibacterial activity. The crude root

extract of *P. sidoides* also showed significant antimycobacterial activity, inhibiting the growth of *Mycobacterium tuberculosis* by 96% at 12.5 μg/ml with an MIC of 100 μg/ml, supporting traditional uses against tuberculosis.

Mativandlela et al. [6] tested *P. sidoides* extracts in ethanol and acetone and found substantial antibacterial effects at high concentrations (5000 mg/L) against respiratory pathogens such as *Haemophilus influenzae*, *Moraxella catarrhalis*, and *Streptococcus pneumoniae*. However, these concentrations were much higher than those required for conventional antibiotics such as streptomycin. In addition, various *P. reniforme* extracts inhibited *M. tuberculosis* at the same concentration, but *P. sidoides* showed no effect against *M. tuberculosis*, indicating lower potency compared to standard treatments.

Lewu et al. [20] showed that acetone and methanol extracts of *P. sidoides* roots and shoots had significant antibacterial activity. MICs ranged from 1 to 5 mg/ml, with the methanol root extract being particularly effective against *Micrococcus kristinae* (MIC 1 mg/ml). These extracts were also effective against respiratory pathogens such as *Bacillus cereu*s and *Klebsiella pneumoniae* but showed limited activity against Gram-negative bacteria such as *Escherichia coli*, *Serratia marcescens*, and *Pseudomonas aeruginosa*, even at the highest concentration (10 mg/ml), underscoring the importance of solvent choice in extraction.

Van Wyngaard et al. [18,44] observed that *P.* s*idoides* root extracts showed different antibacterial activities depending on ethanol concentration. The 11% ethanol extracts had MIC values ranging from 0.313 to 2.5 mg/ml, while the 60% ethanol extracts showed improved efficacy with MIC values ranging from 0.078 to 2.5 mg/ml for Gram-negative bacteria and 0.313 to 2.5 mg/ml for Gram-positive bacteria. Bacteria tested included *Streptococcus pyogenes*, *Proteus mirabilis*, *Staphylococcus aureus*, *Escherichia coli*, *Streptococcus pneumoniae*, *Haemophilus influenzae*, and *Staphylococcus epidermidis*. The increased efficacy of the 60% ethanol extracts was attributed to higher concentrations of umckalin and polyphenols.

In contrast, Mahboubi et al. [44] evaluated ethanolic extracts of *P. graveolens* and *Oliveria decumbens* against clinical *S. aureus* isolates. *P. graveolens* showed significant antimicrobial activity with MIC values ranging from 0.2 to 0.8 g/L, while *O. decumbens* showed MIC values ranging from 9.6 to 38.4 g/L. Bayoub et al. [45] further investigated the antibacterial properties of ethanol extracts of 13 medicinal plants, including *P. graveolens* against *Listeria monocytogenes*. *P. graveolen*s had a MIC of 6.15 mg/ml, while clove and mint timija extracts showed superior efficacy with MIC values of 0.25 mg/ml and 0.315 mg/ml, respectively.

Coronado–López et al. [61] evaluated the antimicrobial activity of methanolic extracts of *P. peltatum* against S*treptococcus mutans* and *Streptococcus sanguinis*. The MIC values for leaf and root extracts against *S. mutans* were 250 mg/ml and for stem extracts 125 mg/ml. For *S. sanguinis*, both leaf and root extracts had MICs of 125 mg/ml. Major phytochemicals identified included flavonoids, tannins, steroids, anthocyanins, quinones, and saponins. Chlorhexidine (CHX), used as a reference, showed superior antibacterial activity with lower MIC values.

Ozbilge et al. [34] evaluated the antimicrobial activity of *P. endlicherianum* root extracts. Using 11% ethanol and 70% methanol extracts, they tested concentrations ranging from 0.375 to 30.00 mg/ml using the agar dilution method. The 70% methanol extract showed the highest antibacterial activity against *Staphylococcus aureus* (MIC 1.38 mg/ml). However, both extracts showed no effect against *Klebsiella pneumoniae* and *Proteus mirabilis*. Significant antibacterial activity was observed against several microorganisms, including *Staphylococcus aureus*, *Streptococcus pyogenes*, and *Escherichia coli*. High levels of phenolic compounds in the methanol extract probably contributed to its potent antibacterial properties.

Table 2 provides a summary of the minimum inhibitory concentrations (MICs) of different crude extracts from various *Pelargonium* plant species, including details on the plant part used and the solvent used for extraction.

Table 2. Minimum inhibitory concentration of different crude extracts from *Pelargonium* plant species.

Pelargonium Source	Plant extract	Part plant	MIC	Bacteria	Reference
P. sidoides	EPS® 7630	Roots	13.8 (mg/mL)	*K. pneumoniae* V 6089	[25]
			3.3 (mg/mL)	*P. mirabilis* ATCC 14153	
			3.3 (mg/mL)	*S. aureus* ATCC 25923	
	EPS® 7630		0.313 (mg/mL)	*E. coli*	[18]
			0.078 (mg/mL)	*P. aeruginosa*	
			0.313 (mg/mL)	*K. pneumoniae*	
			0.313 (mg/mL)	*S. aureus*	
	Ethanolic 60 %		0.313 (mg/mL)	*E. coli*	
			0.156 (mg/mL)	*P. aeruginosa*	
			0.313 (mg/mL)	*K. aerogenes*	
			0.156 (mg/mL)	*S. aureus*	
			0.313 (mg/mL)	*B. cereus*	
			$5x10^3$ (mg/L)	*M. catarrhalis*	
	Ethanolic		$5x10^3$ (mg/L)	*S. pneumoniae*	[6]
			$5x10^3$ (mg/L)	*H. influenzae*	
	Methanolic		5 (mg/mL)	*B. cereus*	[20]
			2.5 (mg/mL)	*S. epidermidis*	
			5 (mg/mL)	*S. aureus*	
			1 (mg/mL)	*M. kristinae*	
			5 (mg/mL)	*S. pyogenes*	
			5 (mg/mL)	*S. pooni*	
		Shoot	5 (mg/mL)	*B. cereus*	
			5 (mg/mL)	*S. epidermidis*	
			5 (mg/mL)	*S. aureus*	
			2.5 (mg/mL)	*M. kristinae*	
			2.5 (mg/mL)	*S. pyogenes*	
			2.5 (mg/mL)	*S. pooni*	
P. reniforme	Ethanolic	Roots	$5x10^3$ (mg/mL)	*M. tuberculosis*	[6]
P. graveolens	Ethanolic 70%	Aerial parts	0.2 – 0.8 (mg/mL)	*S. aureus*	[44]
	Ethanolic 70%	Leaves	6.15 (mg/mL)	*L. monocytogenes.*	[45]
P. peltatum	Methanolic	Leaf	250 (mg/mL)	*S. mutans*	[61]
		Stem	125 (mg/mL)		
		Roots	250 (mg/mL)		
		Leaf	125(mg/mL)	*S. sanguinis*	

		Stem	31 (mg/mL)		
		Roots	25 (mg/mL)		
P. endlicherianum	Ethanolic 11%	Roots	2.83 (mg/mL)	*S. aureus* ATCC 25923	[34]
			12.92 (mg/mL)	*S. pyogenes* ATCC 19615	
			24.17 (mg/mL)	*S. agalactia* ATCC 12401	
			25.00 (mg/mL)	*S. pneumoniae* ATCC 6303	
			23.33 (mg/mL)	*S. mutans* (clinical isolate)	
			24.17 (mg/mL)	*S. sanguinis* DSM 20567	
			9.58 (mg/mL)	*E. faecalis* ATCC 29212	
			28.33 (mg/mL)	*S. epidermidis* (clinical isolate)	
			27.50 (mg/mL)	*Staphylococcus caprae* (clinical isolate)	
			28.33 (mg/mL)	*L. acidophilus* ATCC 11975	
			17.17 (mg/mL)	*E. coli* ATCC 25922	
			7.92 (mg/mL)	*P. aeruginosa* ATCC 27853	
	Methanolic 70%		1.38 (mg/mL)	*S. aureus* ATCC 25923	
			9.17 (mg/mL)	*S. pyogenes* ATCC 19615	
			20 (mg/mL)	*S. agalactia* ATCC 12401	
			24.17 (mg/mL)	*S. pneumoniae* ATCC 6303	
			10.42 (mg/mL)	*S. mutans* (clinical isolate)	
			23.33 (mg/mL)	*S. sanguinis* DSM 20567	
			5.08 (mg/mL)	*E. faecalis* ATCC 29212	
			27.50 (mg/mL)	*S. epidermidis* (clinical isolate)	
			28.33 (mg/mL)	*S. caprae* (clinical isolate)	
			29.17 (mg/mL)	*L. acidophilus* ATCC 11975	
			9.17 (mg/mL)	*E. coli* ATCC 25922	
			4.75 (mg/mL)	*P. aeruginosa* ATCC 27853	
P. odaratissimum	Methanolic 80%	Plant without root	500 (µg/mL)	*S. aureus*	[72]
P. zonale	Ethanolic	Leaves	6.25 (mg/mL)	R. *pseudosolanacearum*	[52]

The initial observation is the extensive range of MIC values. Additionally, it is uncommon to encounter articles utilizing the same plant and target organism, which further complicates a meta-analysis approach. In general, the majority of articles indicate that flavonoids are the primary contributors to the observed antibacterial effects (and coumaric acids in certain instances). However, in all of the analyzed publications, pure compounds were evaluated to confirm the potential chemical responsible for the observed biological activity.

1.3. Anti-inflammatory Activity

Anti-inflammatory activity refers to the capacity of substances or treatments to reduce or modulate the body's inflammatory response. This activity can be achieved through various mechanisms, including the inhibition of pro-inflammatory cytokine production, the blocking of inflammatory enzymes, the modulation of immune cell function, the reduction of oxidative stress, and the interference with inflammatory signaling pathways. Anti-inflammatory agents may also suppress the expression of adhesion molecules, regulate vascular permeability, and influence the balance of lipid mediators. This activity is crucial in managing conditions where inflammation plays a key role, ranging from acute infections to chronic diseases.

Our review indicates that *P. sidoides* has a greater number of studies supporting its anti-inflammatory activity than other species. Nöldner and Schötz [93] demonstrated that the EPS® 7630 extract could counteract lipopolysaccharide (LPS)-induced disease behavior in rats. The authors determined that the high molecular weight fraction (430 kDa) is primarily responsible for this effect, although the molecular basis for these effects is unclear. They suggest that these molecules could be metabolized by intestinal enzymes into smaller, bioavailable entities responsible for the anti-inflammatory activity.

Ref. [26] further evaluated the antitussive, secretolytic, and anti-inflammatory effects of EPS® 7630 in animal models. In a rat model of acute bacterial bronchitis, the researchers measured the histopathological lesions in lung tissue and the serum levels of malondialdehyde (MDA), an indicator of oxidative stress. The results demonstrated that EPS® 7630 significantly reduced lung lesions and MDA levels, suggesting a potent anti-inflammatory and antioxidant effect. Additionally, a positive upregulation of superoxide dismutase (SOD), a key antioxidant enzyme, was observed.

In a more recent study, Van Wyngaard et al. [18] compared the 11% and 60% ethanolic extracts of *P. sidoides* root. The study evaluated the production of the pro-inflammatory cytokine IL-6 and nitric oxide (NO) in LPS-stimulated RAW 264.7 macrophages. Both extracts demonstrated a significant reduction in IL-6 levels, with the 60% extract exhibiting greater efficacy. Additionally, the 60% extract exhibited greater NO inhibition, indicating superior anti-inflammatory activity. The authors propose that the higher efficacy observed in the 60% extract is due to its higher polyphenol content, suggesting that these compounds may be primarily responsible for the observed anti-inflammatory activity.

Another study [35] evaluated the anti-inflammatory effects of *P.* endlicherianum and *P. quercetorum* root extracts in comparison to EPS® 7630 on LPS-stimulated RAW 264.7 macrophages. Extracts of *P. endlicherianum* (11% ethanol and 70% methanol) were observed to significantly inhibit the production of pro-inflammatory cytokines (TNF-α and IL-6), cyclooxygenase-2 (COX-2), and inducible nitric oxide synthase (iNOS), as well as the release of prostaglandin E-2 (PGE2) and NO, without causing cytotoxicity. Moreover, they inhibited the activation of mitogen-activated protein kinase (MAPK) signaling pathways and the nuclear translocation of NF-κB. In contrast, *P. sidoides* alleviated P38/MAPK activation and COX-2 expression, whereas *P.* quercetorum only reduced TNF-α production. These findings highlight the potential of *P. endlicherianum* as an anti-inflammatory agent that exhibits superior efficacy compared to *P. quercetorum an*d EPS® 7630.
